# Sustained attention required for effective dimension-based retro-cue benefit in visual working memory

**DOI:** 10.1167/jov.23.5.13

**Published:** 2023-05-16

**Authors:** Ruyi Liu, Lijing Guo, Hong-jin Sun, Tiina Parviainen, Zifang Zhou, Yuxin Cheng, Qiang Liu, Chaoxiong Ye

**Affiliations:** 1 Institute of Brain and Psychological Sciences, Sichuan Normal University, Chengdu, China; 2 Institute of Brain and Psychological Sciences, Sichuan Normal University, Chengdu, China; 3 Department of Psychology, University of Jyväskylä, Jyväskylä, Finland; 4 Department of Psychology, Neuroscience and Behaviour, McMaster University, Hamilton, Canada; 5 Department of Psychology, University of Jyväskylä, Jyväskylä, Finland; 6 Centre for Interdisciplinary Brain Research, University of Jyväskylä, Jyväskylä, Finland; 7 Institute of Brain and Psychological Sciences, Sichuan Normal University, Chengdu, China; 8 Institute of Brain and Psychological Sciences, Sichuan Normal University, Chengdu, China; 9 Institute of Brain and Psychological Sciences, Sichuan Normal University, Chengdu, China; 10 Institute of Brain and Psychological Sciences, Sichuan Normal University, Chengdu, China; 11 Department of Psychology, University of Jyväskylä, Jyväskylä, Finland; 12 Department of Psychology, Neuroscience and Behaviour, McMaster University ,Hamilton, Canada; 13 Faculty of Social Sciences, Tampere University, Tampere, Finland

**Keywords:** visual short-term memory, retro-cue, dimension, interference, dual task, sustained attention

## Abstract

In visual working memory (VWM) tasks, participants’ performances can be improved through the use of dimension-based retro-cues, which direct internal attention to prioritize a particular dimension (e.g., color or orientation) of VWM representations even after the stimuli disappear. This phenomenon is known as the dimension-based retro-cue benefit (RCB). The present study investigates whether sustained attention is required for the dimension-based RCB by inserting interference or interruption between the retro-cue and the test array to distract attention. We tested the effects of perceptual interference or cognitive interruption on dimension-based RCB when the interference (Experiments 1 and 2 with masks) or interruption (Experiments 3 and 4 with an odd-even task) occurred concurrently with the stages for the maintenance of prioritized information (long cue-and-interference/interruption interstimulus interval, e.g., Experiments 1 and 3) or the deployment of attention (short cue-and-interference/interruption interstimulus interval, e.g., Experiments 2 and 4). Our results demonstrate that perceptual interference or cognitive interruption attenuates the dimension-based RCB. These findings suggest that sustained attention is necessary for the effective prioritization of a specific dimension of VWM representations.

## Introduction

Visual working memory (VWM) is a transient system that stores and processes visual information from the outside world to achieve certain goals. This type of memory is responsible for allowing individuals to maintain visual information over a short period of time for use in a wide array of cognitive functions (Baddeley, 2012; Cowan, 2001), ranging from trans-saccadic perception (Hollingworth, Richard, & Luck, 2008) to higher cognition (e.g., fluid intelligence, see Luck & Vogel, 2013) and decision making (Xie, Campbell, & Zhang, 2020). The capacity of VWM is limited; typically it can be inaccurate when more than three or four items are maintained (Bays & Husain, 2008; Lewis‐Peacock, Kessler, & Oberauer, 2018; Luck & Vogel, 1997; Ozimič & Repovš, 2020; Schneegans, Taylor, & Bays, 2020; Vogel, Woodman, & Luck, 2001; Ye, Zhang, Liu, Li, & Liu, 2014; Zhang & Luck, 2011) and has attracted the attention of many researchers who seek to improve the efficient allocation of resources within this system (Gao, Gao, Tang, Shui, & Shen, 2016; Hitch, Allen, & Baddeley, 2019; Liu, Liu, Guo, Astikainen, & Ye, 2022; Peterson, Gozenman, Arciniega, & Berryhill, 2015; Treisman & Zhang, 2006; Vogel et al., 2001).

Previous research has found that VWM can focus biased attention on task-relevant information, leading to a rearrangement of VWM resources among representations and thereby compensating for the limited capacity (Liesefeld, Liesefeld, & Zimmer, 2014; Maniglia & Souza, 2020; Plebanek & Sloutsky, 2019; Ye et al., 2018). This phenomenon occurs even when visual stimuli are no longer present in the field of vision (Griffin & Nobre, 2003; Kuo, Yeh, Chen, & D'Esposito, 2011; Landman, Spekreijse, & Lamme, 2003; Matsukura, Cosman, Roper, Vatterott, & Vecera, 2014; Matsukura, Luck, & Vecera, 2007; Matsukura & Vecera, 2015; Murray, Nobre, Clark, Cravo, & Stokes, 2013; Myers, Walther, Wallis, Stokes, & Nobre, 2015; Niklaus, Singmann, & Oberauer, 2019; Pertzov, Bays, Joseph, & Husain, 2013; Souza & Oberauer, 2016). In a typical retro-cue experiment, participants are shown a memory array and asked to remember it for later recall. After a delay, a retro-cue is presented to indicate which item from the memory array is most likely to be tested. Griffin and Nobre (2003) found that the accuracy of recall was higher in valid-cue trials, in which the retro-cue correctly indicated the location of the to-be-tested item, than in neutral trials, where the retro-cue did not provide any useful information. This suggests that the retro-cued representation of an object is preferentially retained. This phenomenon is known as the object-based retro-cue benefit (RCB). The object-based RCB is stable across different stimulus types (Griffin & Nobre, 2003; Landman et al., 2003), spatial configurations (Fu et al., 2022; Kuo, Stokes, & Nobre, 2012), timing parameters during the maintenance interval (Makovski, Sussman, & Jiang, 2008; Schneider, Mertes, & Wascher, 2016; Souza, Rerko, & Oberauer, 2016), and various VWM tasks (Arnicane & Souza, 2021; Griffin & Nobre, 2003). The prioritization of retro-cued object representations in VWM has also been supported by evidence from ERP research, which has shown a smaller amplitude for the contralateral delay activity (an EEG component that decreases in amplitude as the number of representations currently being held in VWM decreases) after a valid retro-cue than after a neutral one (Kuo et al., 2012). In summary, numerous previous studies have concluded that the object-based RCB reflects the internal focus of attention on and the enhancement or protection of the cued object in VWM (Souza & Oberauer, 2016).

In addition to research investigating the mechanism of object-based RCB, recent studies have demonstrated that internal attention can also facilitate behavior when directed toward a shared dimension (such as color or orientation) among multiple items in VWM. These studies, which have used multi-dimensional stimuli, have reported a dimension-based RCB, in which VWM performance is better for retro-cued dimensions than for non-cued ones (Hajonides, van Ede, Stokes, & Nobre, 2020; Heuer & Schubo, 2016; Niklaus, Nobre, & van Ede, 2017; Park, Sy, Hong, & Tong, 2017; Ye, Hu, Ristaniemi, Gendron, & Liu, 2016; Ye et al., 2021).

Although both the object-based and dimension-based retro-cue benefits involve attention processes, each may depend on different mechanisms. Object-based retro-cues may enable individuals to reduce multiple VWM representations to one representation (the cued item), thereby reducing memory load (Kuo et al., 2011; Lepsien, Thornton, & Nobre, 2011; Nobre, Griffin, & Rao, 2007; Souza, Rerko, & Oberauer, 2014). By contrast, dimension-based retro-cues may not reduce the total number of VWM representations, but they can reduce the amount of information that needs to be maintained in each representation (Hajonides et al., 2020; Niklaus et al., 2017). Overall, although early studies largely focused on object-based retro-cues and their influence on VWM representations, more recent research has started to investigate dimension-based RCB. Further study is needed to develop a full understanding of the mechanisms and factors that influence the dimension-based RCB.

In previous research using retro-cue tasks, one direction of study has been to explore how perceptual interference or cognitive interruptions, which compulsively disrupt sustained attention, affect object-based RCB to provide a better understanding of the contribution of sustained attention to object-based RCB (Hollingworth & Maxcey-Richard, 2013; Makovski & Jiang, 2007; Makovski & Pertzov, 2015; Makovski et al., 2008; Matsukura et al., 2007; Pertzov et al., 2013; Pinto, Sligte, Shapiro, & Lamme, 2013; Rerko, Souza, & Oberauer, 2014; Souza, Rerko, & Oberauer, 2016; van Moorselaar, Theeuwes, & Olivers, 2014). For example, in a recall task with retro-cues, van Moorselaar et al. (2014) introduced masks (perceptual interference) in the post-cue interval to disrupt the sustained attention directed by retro-cues. When the cue-and-interference stimulus-onset asynchrony (SOA) was short, perceptual interference weakened the object-based RCB. However, when participants were given sufficient time (500–600 ms) to shift their attention, the object-based RCB was not affected by the perceptual interference. These findings suggest that the necessity for sustained attention depends on the temporal course of retro-cue use. Specifically, sustained attention is required for the deployment of attention to the task-relevant representation within a short delay (about 500 ms) after the onset of a valid retro-cue. However, when the undisrupted time to use a retro-cue is sufficient for VWM resources reallocation, diverting attention does not impair the maintenance of prioritized information and a robust object-based RCB is preserved.

In addition to using stimulus-driven interference, such as masks, researchers have also used attention-demanding interruption tasks (cognitive interruption) as top-down interruption to examine the role of sustained attention in the object-based RCB. These tasks involve the insertion of an attention-demanding interruption between the retro-cue and the test array. For instance, Hollingworth and Maxcey-Richard (2013) used an attention search task, Rerko, Souza, and Oberauer (2014) used a visual classification task, and Makovski and Pertzov (2015) used an odd-even interruption task to redirect participants' attention away from the cued item. These previous studies have indicated that the object-based RCB is not diminished by attention-demanding tasks, suggesting that the maintenance of the object-based RCB may not require sustained attention (Hollingworth & Maxcey-Richard, 2013; Rerko et al., 2014). One important point to note is that additional research is needed for a full understanding of the relationship between sustained attention and the RCB, and the findings of these studies should be considered within the context of the experimental designs used.

To the best of our knowledge, the current literature lacks research on the impact of perceptual interference or attention-demanding interruptions on the dimension-based RCB, and this has hindered our understanding of the role of sustained attention in the dimension-based RCB. Notably, with object-based retro-cues, participants can use the cues to selectively focus their attention on a single cued item, thereby enhancing the maintenance or reducing the decline of the cued representation. However, when using dimension-based retro-cues, participants must distribute their attention among all items, potentially leading to differences in the effect of interference on the dimension-based RCB versus the object-based RCB. The dimension-based RCB may possibly require sustained attention; therefore perceptual interference or attention-demanding interruptions may attenuate it.

The aim of the present study was to investigate the impact of perceptual interference and attention-demanding interruptions on the dimension-based RCB to determine the role of sustained attention in the dimension-based RCB. The research question was addressed by four experiments. Experiment 1 examined the effect of perceptual interference, which was presented in the stage for the maintenance of prioritized information (i.e., with a long cue-and-interference interstimulus interval [ISI]), on the dimension-based RCB. Experiment 2 investigated the influence of perceptual interference that occurred in the stage involving the deployment of attention directed by a retro-cue (i.e., with a short cue-and-interference ISI), on the dimension-based RCB. Experiment 3 assessed the effect of an attention-demanding interruption task, inserted in the stage for the maintenance of prioritized information, on the dimension-based RCB. Experiment 4 explored the influence of an attention-demanding interruption task, displayed in the stage involving the deployment of attention directed by a retro-cue, on the dimension-based RCB. If the dimension-based RCB does not require sustained attention, we predicted that the dimension-based RCB would not be affected by interference or interruption, resulting in no significant difference in the dimension-based RCB under the interference/interruption and the no-interference/no-interruption conditions. Conversely, if the dimension-based RCB requires sustained attention, we anticipated that the presence of interference or interruption would attenuate the dimension-based RCB, resulting in a significantly smaller dimension-based RCB under the interference/interruption condition than under the no-interference/no-interruption condition. The results of this study provide some new insights into the relationship between sustained attention and the dimension-based RCB and the cognitive mechanisms underlying the dimension-based RCB.

## Experiment 1

Before Experiment 1, we conducted a pilot experiment that used a change detection task with perceptual interference (masks) to explore the research question. However, no significant effects of perceptual interference were observed (see the Supplementary Materials for more details). This may reflect the fact that, in the change detection task, the participants were only required to memorize low-precision memory representations to complete the task; therefore the task results were insensitive to interference from the masks. Consequently, to address our research question, we exclusively used a recall task that necessitates high-precision maintenance of VWM representations; this task has been previously used to investigate dimension-based representations in VWM (Hajonides et al., 2020; Heuer & Schubo, 2016; Niklaus et al., 2017; Park et al., 2017; Ye et al., 2016; Ye et al., 2021).

In Experiment 1, we investigated the effect of perceptual interference on dimension-based RCB in VWM, while allowing participants sufficient time to shift their attention to the relevant information before the diversion of attention. To achieve this, we introduced masks at a long cue-and-interference SOA to disrupt sustained attention and fixed the ISI between cue offset and mask onset at 1000 ms in the mask condition, which is consistent with previous studies by Makovski and Pertzov (2015). Furthermore, to align with recent research on dimension-based RCB (Hajonides et al., 2020), we used a memory load of two items, each comprising two features.

### Method

#### Participants

Based on the previous study by Hajonides et al. (2020) on dimension-based retro-cues, we predicted a similar effect size ($\eta_{p}^{2}$ = 0.394) for our experimental design. A power analysis using G*Power 3.1.9.2 (Faul, Erdfelder, Lang, & Buchner, 2007) indicated that 12 participants were needed to achieve 95% power at an alpha level of 0.05. We recruited 24 participants for Experiment 1, following the study by van Moorselaar et al. (2014) on the perceptual interference effect on the object-based RCB (n = 24), to ensure an adequate sample size. These participants were college or postgraduate students (19 females and five males; 19.08 ± 1.02 years old, age range 18–22 years; all right-handed) who reported normal or corrected-to-normal vision and no history of neurological problems. They provided written informed consent and received monetary compensation for their participation. Our study was approved by the ethical committee of Sichuan Normal University and followed the guidelines of the Declaration of Helsinki (2008).

#### Materials and apparatus

The recall task had four stages: the memory array, cue, mask array, and test array. We generated a pool of 180 orientations and 360 colors using the same method as in the previous study by Ye et al. (2016). The visual stimuli were colored bars (1.1° long and 0.4° high) presented on a gray (128, 128, 128) background. The color and orientation of each memory stimulus were randomly chosen from the 360 available colors (ranging from 1 to 360 in one-color step increments; see the Supplementary Materials for more details) and the 180 available orientations (ranging from 1 to 180 in one-step increments). The two bars differed by at least 30° orientation and 60 color steps. The bars were located 1.5° to the left and right of the central fixation point. The dimension-based retro-cues were the words “color” and “orientation,” which indicated to the participants which of these two dimensions would be tested. The neutral retro-cue was the word “all,” which indicated that either of the two dimensions could be tested. All words were presented in Chinese and were displayed in the center of the screen. The mask array consisted of eight intertwined colored bars (1.8° × 0.4°) with different orientations, spaced 45° apart and selected from the pool of memorandum colors. A total of 80 mask patterns were randomly generated. The test array for the color recall task consisted of an outlined square and a color wheel (5.8° inner radius, 2.2° thickness). The test array for the orientation recall task consisted of an outlined square (1.2° × 1.2°) and a vertical white bar (1.1° × 0.4°) displayed at fixation. The entire experiment was conducted in a softly lit, soundproof room using 19-inch screens (1280 × 768), with the participant seated approximately 60 cm away.

#### Procedure

**Figure 1.**
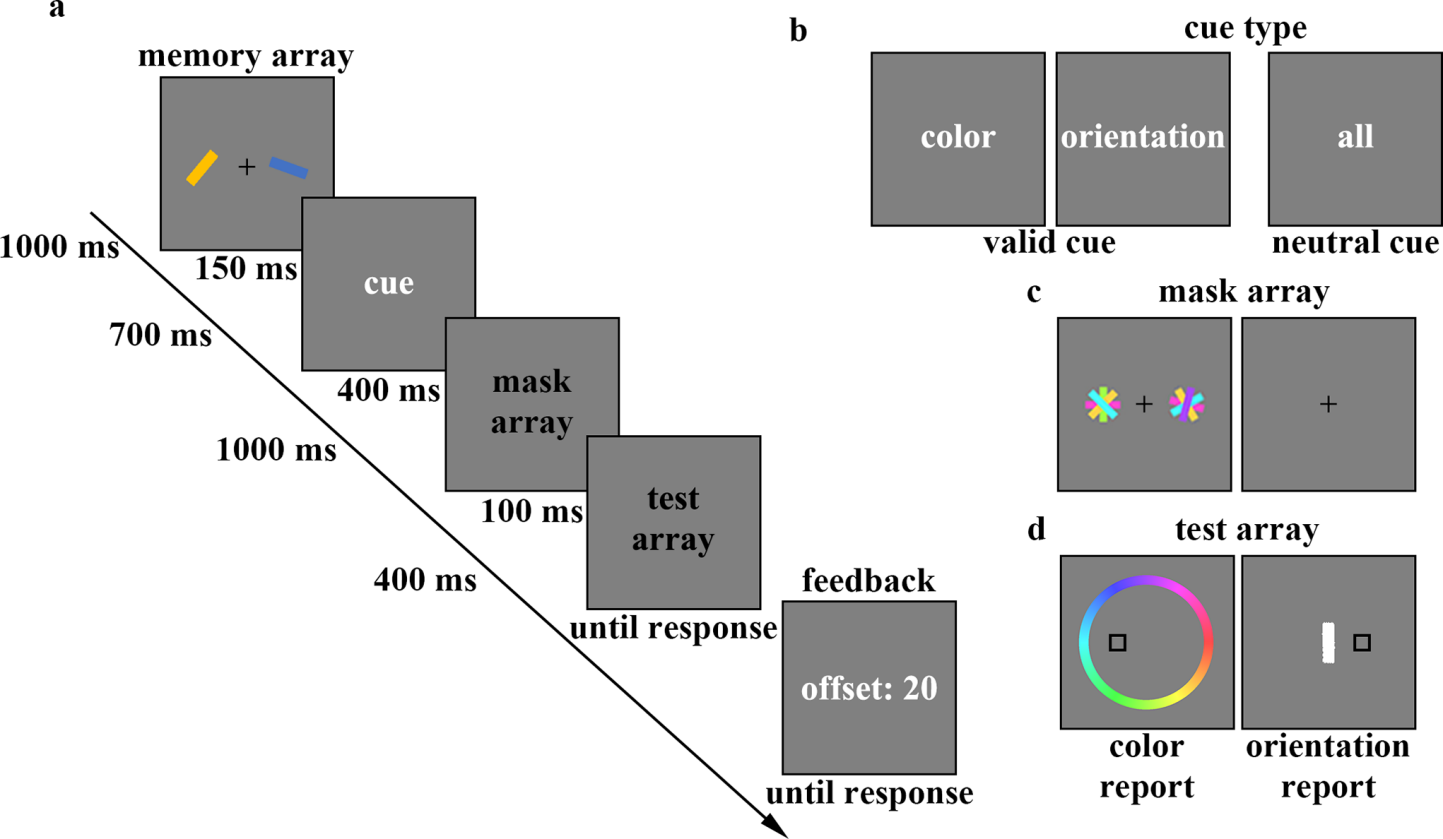
The flowchart of a recall task in Experiment 1 (a). Half the trials contained neutral cues showing the word “all” (in Chinese) and the other half displayed valid cues, words implying the to-be-tested dimension (b). In the mask array, we provided two masks on the site of the memoranda (c, left) or nothing but a fixation (c, right), with equal probability. When the color was probed, a color wheel appeared with options (d, left), and when the orientation of the target bar was to be reported, a white bar appeared at the center of the screen (d, right).

The procedure for Experiment 1 is illustrated in Figure 1. At the beginning of each trial, a central fixation point (a black cross) was displayed on the center of the screen for 1000 ms, and participants were instructed to keep their eyes on the fixation point throughout the experiment. The memory array was then presented for 150 ms, and participants were asked to remember the color and orientation of the two bars on the screen. After a 700 ms blank period, the retro-cue was presented for 400 ms. Half of the trials included a 100% valid cue that indicated which dimension (color or orientation) would be tested, while the other half included a neutral cue. Following the cue and a 1000 ms blank period, under the mask condition, two masks with irrelevant visual information were presented for 100 ms on the site of the memoranda to interfere with attention focused on the cued representations. Half of the trials contained masks (mask condition), whereas the other half only displayed the fixation point for 100 ms (no-mask condition). After a 400 ms blank period, a black square was presented on the site of the probed memory stimulus during the test array. In the test array, the report type was randomly selected for each trial. For color report trials, a color wheel was centered on the fixation and consisted of 360 colored segments corresponding to possible stimulus colors. Participants were asked to report the color of the stored item at the location of the black square outline by clicking the left mouse button when the cursor was positioned on the targeted color at the wheel. For orientation report trials, an adjustable vertical white bar was presented at the fixation point. Participants adjusted the white bar's orientation to match that of the cued bar by moving the cursor with the mouse and pressing the left mouse button to rotate the white bar to the cursor position. Once satisfied, they pressed the right mouse button to finalize their response. There were no time constraints on responses. The test array remained on the screen until the response was given, after which feedback on performance offset (measured by the deviation of the participant's response from the target stimulus value) was provided.

We used 100 trials for each condition (valid cue–mask–color, valid cue–no mask–color, neutral cue–mask–color, neutral cue–no mask–color, valid cue–mask–orientation, valid cue–no mask–orientation, neutral cue–mask–orientation, neutral cue–no mask–orientation), for a total of 800 trials. The trials were fully randomized. The experiment lasted approximately 60 minutes, including instructions and at least 16 practice trials before the main task. Participants were given a short break every 80 trials.

#### Data analysis

We calculated the errors for each participant and each experimental condition by subtracting the value of the probed item from the response. The main dependent variable was the absolute value of the deviation, which we referred to as the offset. Note that the offset depends on the defined color step or orientation degree. Because the response ranges for color (1–360 color steps) and orientation (1–180 orientation degrees) differ, a larger offset in the color report trials does not necessarily indicate worse color memory performance compared to the orientation memory performance. The qualitative differences in the color and orientation reports impelled us to conduct separate analyses for the offsets in the color and orientation report trials. We investigated the influence of interfering information on the use of dimension-based retro-cues by conducting repeated measures analysis of variance (ANOVA) for the offsets with different cue types (neutral cue vs. valid cue) and interference condition (mask vs. no mask) as within-subject factors. We conducted two-tailed t-tests for follow-up comparisons between different conditions. A significance level of *p* < 0.05 was used for all tests. The value of $\eta_{p}^{2}$ was used as an estimator of the effect size for ANOVA, and Cohen's *d* was used as an estimator of the effect size for *t*-tests. We also used JASP (version 0.17.1, JASP Team, 2023) to conduct a Bayes factor analysis to report the *t*-test results. The results of Bayes factors show whether the *t*-test results supported the alternative hypothesis or null hypothesis (Rouder, Speckman, Sun, Morey, 2009; Schmalz, Biurrun Manresa, & Zhang, 2021), thereby providing an odds ratio for the alternative/null hypotheses (values <1 favor the null hypothesis, and values >1 favor the alternative hypothesis). The default priors in JASP were used. The *p* values of the follow-up comparisons were corrected using the Bonferroni method and are denoted as *p*_bonf_. We also reported the circular standard deviation of errors (SD_error_) as an overall measure of memory error in the recall task. The results of SD_error_ were consistent with those of the offset; therefore we provided those results in the Supplementary Materials.

We directly evaluated the impact of masks on dimension-based RCB by calculating a retro-cue benefit index (RBI), which reflected the relative improvement between the valid- and neutral-cue conditions. The definition of the RBI is as follows:

$$\mathrm{RBI}=\frac{offset_{neutral}-offset_{valid}}{offset_{neutral}}.$$

The RBI was then compared to a constant, zero, using one-sample *t*-tests across the mask and no-mask conditions. We expected an RBI that exceeded zero as an illustration of the dimension-based RCB. Conversely, an RBI ≤0 indicated that the participants performed VWM tasks no better in valid-cue trials than in neutral-cue trials. We also conducted a paired-samples *t*-test to compare the RBIs between interference conditions to examine the effect of interference on the dimension-based RCB. If no difference was found in the RBIs between the mask and no-mask conditions, we inferred that the RCB was independent of the interference. However, if the RBI was smaller in trials with masks than without masks, this was considered an indication that the interference impaired the dimension-based RCB. We used Cohen's *d* and Bayes factor analysis to report the *t*-test results.

We also used the mixture model (Zhang & Luck, 2008) and swap model (Bays, Catalao, & Husain, 2009) to analyze the offset data using the MemToolbox (Suchow, Brady, Fougnie, & Alvarez, 2013); these data are presented in the Supplementary Materials. However, an important point to note is that these models have been challenged by recent research (Schneegans et al., 2020; Schurgin, Wixted, & Brady, 2020; van den Berg, Shin, Chou, George, & Ma, 2012). Therefore we acknowledge that the results of model fitting should be interpreted with caution. As such, we primarily used the mean offset and RBI values as the main dependent variables to evaluate the VWM performance of participants. We further supported our findings from the color report trials by conducting supplementary analyses to show that participants remembered the continuous color values, not just the color categories (see Supplementary Materials).

### Results

#### Offset

**Figure 2.**
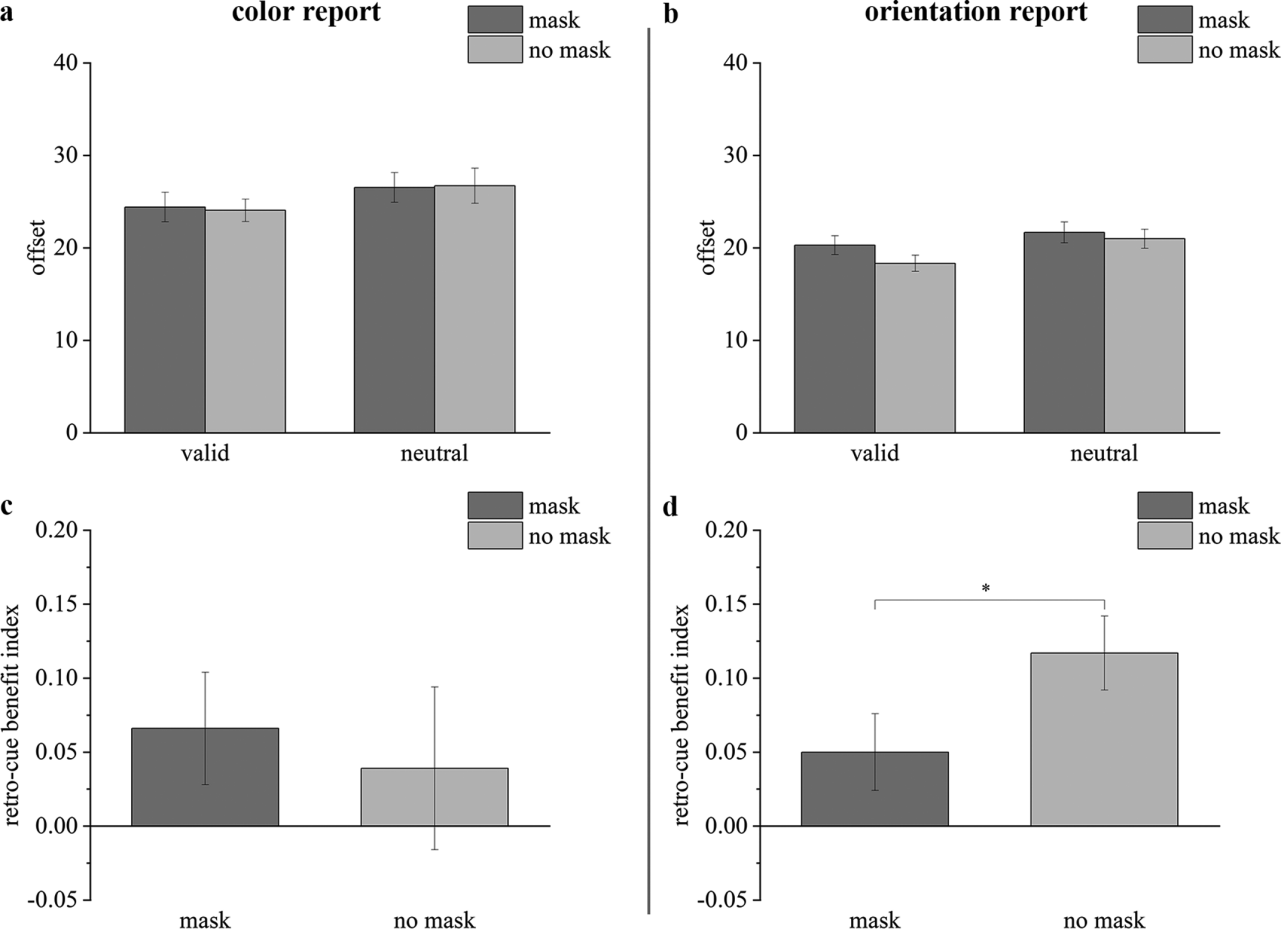
Results of Experiment 1 for mean offsets (a: color report trials; b: orientation report trials) and RBIs (c: color report trials; d: orientation report trials). The dark gray bars represent results in the mask condition; the light gray bars represent results in the no-mask condition. * *p* < 0.05. Error bars reflect within-subject SEMs.

The results of Experiment 1 are shown in Figure 2, which presents the offset for each condition for the color report trials (Figure 2a) and the orientation report trials (Figure 2b). For the color report trials, the ANOVA showed no significant interaction between the cue type and interference condition (*F*(1, 23) = 0.107, *p* = 0.747, $\eta_{p}^{2}$ = 0.005). The interference condition showed no significant main effect (*F*(1, 23) = 0.011, *p* = 0.916, $\eta_{p}^{2}$ < 0.001), but a significant main effect was detected for the cue type (*F*(1, 23) = 5.561, *p* = 0.027, $\eta_{p}^{2}$ = 0.195). Follow-up comparisons revealed that the offset was significantly smaller under the valid-cue trials than under the neutral-cue trials for the mask condition (*t*(23) = 2.106, *p*_bonf_ = 0.046, Cohen's *d* = 0.269, *BF*_10_ = 1.382). By contrast, no significant difference was detected between the valid-cue trials and the neutral-cue trials for the no-mask condition (*t*(23) = 1.703, *p*_bonf_ = 0.102, Cohen's *d* = 0.241, *BF*_10_ = 0.752). Additionally, no significant difference was detected between the mask and no-mask conditions for either the valid-cue trials (*t*(23) = 0.368, *p*_bonf_ = 0.716, Cohen's *d* = 0.075, *BF*_10_ = 0.228) or the neutral-cue trials (*t*(23) = 0.156, *p*_bonf_ = 0.877, Cohen's *d* = 0.023, *BF*_10_ = 0.217).

For the orientation report trials, the ANOVA identified a significant interaction between the cue type and interference condition (*F*(1, 23) = 5.406, *p* = 0.029, $\eta_{p}^{2}$ = 0.190) and significant main effects for the cue type (*F*(1, 23) = 14.962, *p* = 0.001, $\eta_{p}^{2}$ = 0.394) and the interference condition (*F*(1, 23) = 13.724, *p* = 0.001, $\eta_{p}^{2}$ = 0.374). Follow-up comparisons revealed that the offset was significantly smaller under the valid-cue trials than under the neutral-cue trials for both the mask condition (*t*(23) = 2.203, *p*_bonf_ = 0.038, Cohen's *d* = 0.450, *BF*_10_ = 1.621) and the no-mask condition (*t*(23) = 4.811, *p*_bonf_ < 0.001, Cohen's *d* = 0.982, *BF*_10_ = 352.262). A significant difference was detected between the mask and no-mask conditions for the valid-cue trials (*t*(23) = 5.352, *p*_bonf_ < 0.001, Cohen's *d* = 1.093, *BF*_10_ = 1180.574) but not for the neutral-cue trials (*t*(23) = 1.314, *p*_bonf_ = 0.202, Cohen's *d* = 0.268, *BF*_10_ = 0.460).

#### RBI

The average RBIs are also illustrated in the color report trials (Figure 2c) and the orientation report trials (Figure 2d). For the color report trials, the one sample *t*-test revealed that the RBI in the mask conditions was significantly larger than 0 (*t*(23) = 1.744, *p*_bonf_ = 0.047, Cohen's *d* = 0.356, *BF*_10_ = 1.505), but the RBI in the no-mask conditions was not significant different from 0 (*t*(23) = 0.714, *p*_bonf_ = 0.241, Cohen's *d* = 0.146, *BF*_10_ = 0.405). The paired-samples *t*-test did not reveal significant differences in RBI between the mask condition and no-mask condition (*t*(23) = 0.430, *p*_bonf_ = 0.664, Cohen's *d* = 0.088, *BF*_10_ = 0.160).

For the orientation report trials, the one sample *t*-test revealed that the RBI was significantly larger than 0 both in the mask conditions (*t*(23) = 1.904, *p*_bonf_ = 0.035, Cohen's *d* = 0.389, *BF*_10_ = 1.928) and in the no-mask conditions (*t*(23) = 4.691, *p*_bonf_ < 0.001, Cohen's d = 0.958, *BF*_10_ = 539.030). The paired-samples *t*-test revealed a significantly smaller RBI in the mask condition than the no-mask condition (*t*(23) = 2.498, *p*_bonf_ = 0.010, Cohen's *d* = 0.510, *BF*_10_ = 5.358).

### Discussion

In Experiment 1, we examined the effects in the color report trials and orientation report trials separately. Our results revealed distinct patterns for the color report trials and the orientation report trials.

In the orientation report trials, the offsets were smaller in valid-cue trials than in neutral-cue trials, regardless of the presence of masks after the retro-cue. This finding supports the existence of a dimension-based RCB, which is consistent with previous literature in the field (Hajonides et al., 2020; Heuer & Schubo, 2016; Niklaus et al., 2017; Park et al., 2017; Ye et al., 2016; Ye et al., 2021). We also found that the RBI value was significantly larger for participants under trials without masks than under trials with masks. This result provides evidence that perceptual interference during the stage for the maintenance of prioritized information can weaken the dimension-based RCB. This effect may reflect the fact that sustained attention during stage for the maintenance of prioritized information can facilitate a robust dimension-based RCB, whereas the presence of perceptual interference impairs this sustained attention and leads to an impairment of the dimension-based RCB. However, we should note that while the presence of perceptual interference only weakens the dimension-based RCB, the RCB still exists. This indicates that sustained attention during the stage for the maintenance of prioritized information may maximize the dimension-based RCB, but it is not a necessary factor for its existence.

In the color report trials, we found a significant dimension-based RCB only under the mask condition. However, no statistically significant dimension-based RCB was evident under the no-mask condition, although the performance was numerically better under the valid cue condition than under the neutral cue condition. The RBI value did not differ significantly between the mask and no-mask conditions, suggesting that the dimension-based RCB obtained by participants in the color report trials is relatively weak.

One possible explanation for this weak dimension-based RCB in the color report trials is that maintaining color representations in VWM is a relatively simple task when the memory load is two items. Previous research has found that the average memory capacity for colors in VWM is 2.9 (Vogel & Awh, 2008). In the color report trials, the effort required to use the retro-cue may outweigh its benefits. The participants may maintain the two color representations nearly perfectly, which may lead to a ceiling effect of the VWM performance under both the valid cue and neutral cue conditions, thereby reducing the benefits obtainable from the dimension-based retro-cue by the participants in the color report trials.

By contrast, previous studies have indicated a greater difficulty in remembering orientation stimuli than color stimuli in VWM tasks (Becker, Miller, & Liu, 2013; Park et al., 2017; Stevanovski & Jolicoeur, 2011; Ye et al., 2021). This may explain why we observed a significant dimension-based RCB in the orientation report trials but not in the color report trials. Maintaining the same number of orientations in VWM may demand more attention resources than colors, which might have contributed to the interference effect observed during the stage for the maintenance of prioritized information. The perceptual interference may have captured part of the attention resources, leaving insufficient resources for maintaining cued orientation representations, but it had no significant influence on the cued color representations.

In summary, the findings of Experiment 1 indicate that the presence of perceptual interference can attenuate the dimension-based RCB even when there is an adequate duration of undisturbed time available to use the retro-cue. This attenuation effect is more pronounced in the orientation report trials.

## Experiment 2

Previous research on the object-based RCB has shown that perceptual interference displayed after a long delay (longer than 500–600 ms) after the onset of a retro-cue has little to no effect on the object-based RCB (Pinto et al., 2013; van Moorselaar et al., 2014). Nonetheless, when the cue-and-interference SOA is less than about 500 ms, perceptual interference can attenuate the object-based RCB, with the magnitude of this attenuation rising as the cue-and-interference SOA is shortened. That is, after the appearance of retro-cue, participants need time to reallocate attention, and in this stage involving the deployment of attention, the appearance of interference can cause more harm to the object-based RCB. As such, the effect of perceptual interference on the object-based RCB varies depending on the onset of attention disruption. In Experiment 1, we investigated the influence of perceptual interference on the dimension-based RCB, with a sufficient duration between the cue and masks to utilize a valid retro-cue effectively. In Experiment 2, we sought to explore the effect of perceptual interference on the dimension-based RCB when the undisturbed using time of a retro-cue was short.

In Experiment 2, we manipulated the cue-and-interference SOA to 400 ms and varied eight conditions (valid cue–mask–color, valid cue–no mask–color, neutral cue–mask–color, neutral cue–no mask–color, valid cue–mask–orientation, valid cue–no mask–orientation, neutral cue–mask–orientation, neutral cue–no mask–orientation), similar to Experiment 1. We avoided a ceiling effect due to a low memory load by setting the memory load to 3 items, in accordance with previous research on the dimension-based RCB (Niklaus et al., 2017; Ye et al., 2016; Ye et al., 2021). In addition, inspired by van Moorselaar et al., (2014; Experiment 2), we enhanced the mask effectiveness by adopting flickering masks rather than static masks as the perceptual interference.

Furthermore, recent research by Luo, Huang, & Tian (2023) suggests that the RCB is effective only when VWM consolidation time is inadequate. Our previous research has shown that participants experience two different stages during VWM consolidation, and the resource allocation in these stages is influenced by the sufficiency of VWM consolidation time (Ye et al., 2017; Ye et al., 2020; Ye et al., 2019). Additionally, previous studies indicate that VWM consolidation of orientation stimuli is a serial process that takes about 100 ms to complete for each orientation stimulus (Becker et al., 2013; Hao, Becker, Ye, Liu, & Liu, 2018; Liu & Becker, 2013; Miller, Becker, & Liu, 2014). Therefore, in Experiment 2, we chose a stimulus presentation time of 150 ms to limit VWM consolidation and ensure a more stable RCB.

### Method

#### Participants

Using the same reasoning as in Experiment 1, we ensured a sufficient sample by recruiting a new sample of 23 participants (18 females and five males; 21.30 ± 3.31 years old, age range 17–33 years; two were left-handed). All participants were college or postgraduate students; all had normal or corrected-to-normal vision, and none had a history of neurological issues. They provided written informed consent and received monetary compensation for their participation. Our study received ethical approval from the ethical committee of Sichuan Normal University and followed the guidelines outlined in the Declaration of Helsinki (2008).

#### Materials, apparatus, and procedure

**Figure 3.**
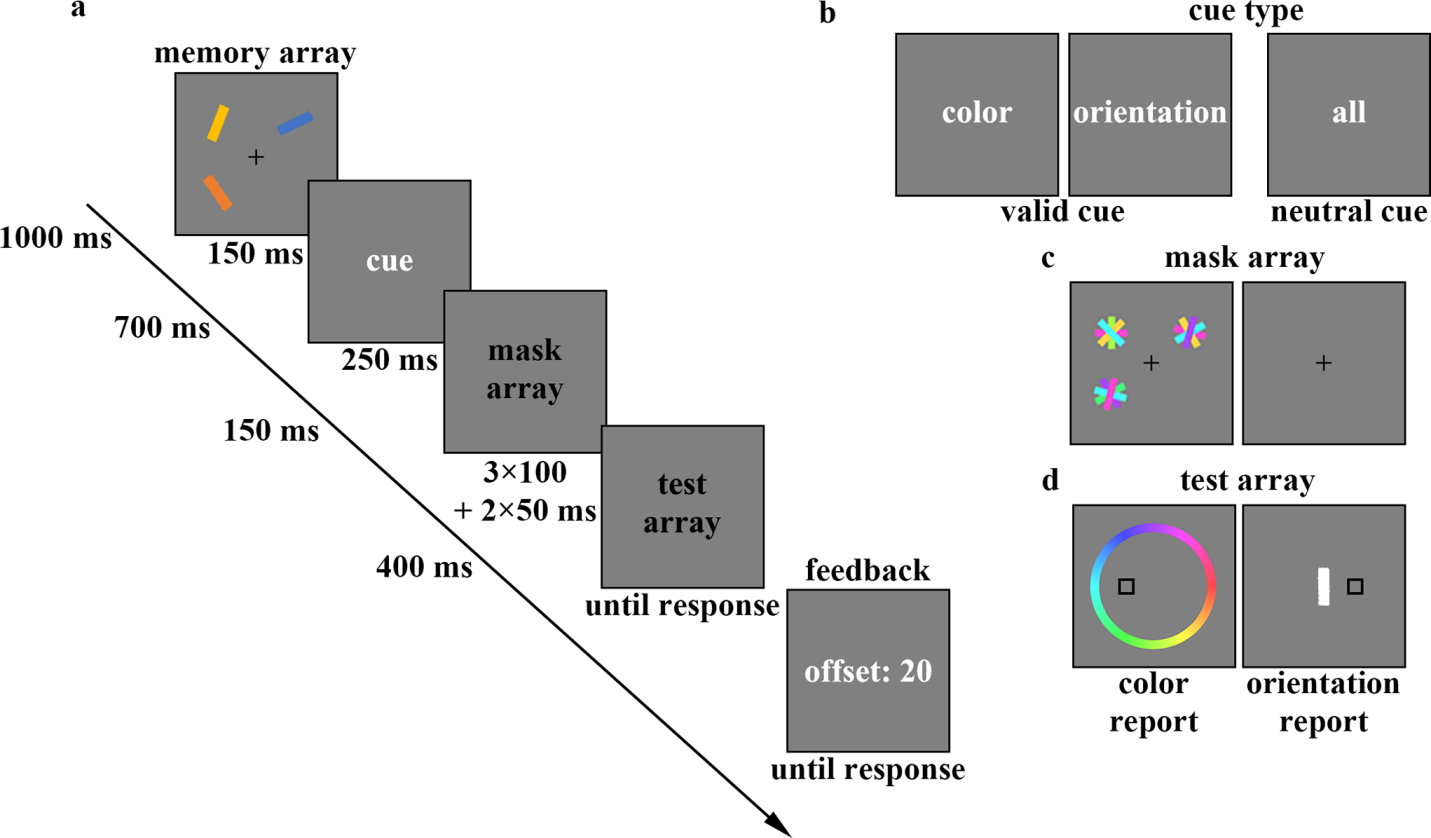
The flowchart of a recall task in Experiment 2 (a). Half the trials contained neutral cues showing the word “all” (in Chinese) and the other half displayed valid cues, which were words implying the to-be-tested dimension (b). In the mask array, we provided three masks on the site of the memoranda (c, left) or nothing but a fixation (c, right), with the equal probability. When the color was probed, a color wheel appeared with options (d, left) and when the orientation of target bar was to be reported, a white bar appeared in the center of the screen (d, right).

The materials and apparatus used in Experiment 2 were the same as in Experiment 1. The procedures of Experiment 2 (shown in Figure 3) were also the same as in Experiment 1, with the following changes: (1) The gap between the retro-cue and mask was shortened by reducing the ISI between the cue and the mask array from 1000 ms to 150 ms. (2) The presentation time of the retro-cue was shortened to 250 ms. (3) The mask array was modified. In the mask condition trials, the mask array was flickering masks, which were presented three times, each lasting for 100 ms, with a blank interval of 50 ms between each presentation. In the no-mask condition trials, the ISI between the cue and the test array was reduced to 950 ms. (4) The memory load was increased to three. In each trial, three colored bars were presented in three randomly chosen locations out of four possible locations, located at the corners of an imaginary square (eccentricity, 2.5°). (5) The number of trials was adjusted. The number of trials in each condition (valid cue–mask–color, valid cue–no mask–color, neutral cue–mask–color, neutral cue–no mask–color, valid cue–mask–orientation, valid cue–no mask–orientation, neutral cue–mask–orientation, neutral cue–no mask–orientation) was reduced to 64. This resulted in a total of 512 trials, which were fully randomized. The experiment lasted approximately 40 minutes and included instructions and at least 16 practice trials before the main task.

#### Data analysis

The data analysis for Experiment 2 was conducted following the same procedure described for Experiment 1, with the exception that we removed the mixture model and swap model fitting because of an insufficient number of trials per condition to support model fitting.

### Results

#### Offset

**Figure 4.**
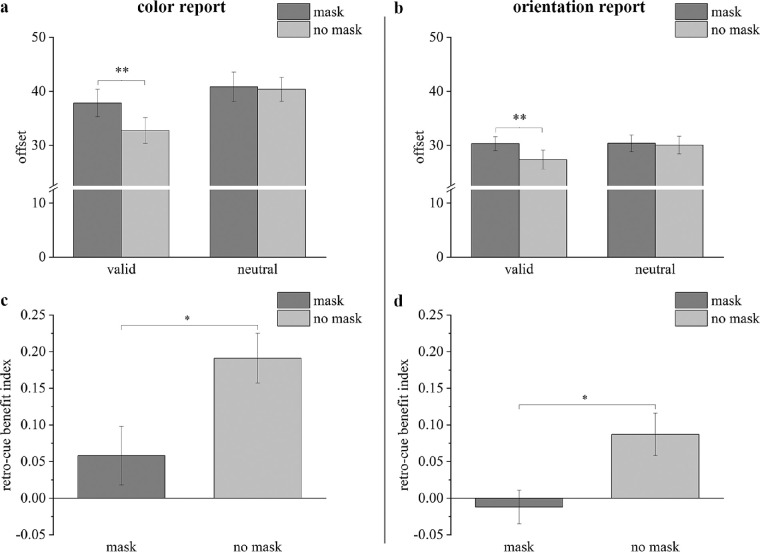
Results of Experiment 2 for mean offsets (a: color report trials; b: orientation report trials) and RBIs (c: color report trials; d: orientation report trials). The dark gray bars represent results in the mask trials; the light gray bars represent results in the no-mask trials. * *p* < 0.05, ** *p* < 0.01. Error bars reflect within-subject SEMs.

The results for Experiment 2 are shown in Figure 4, which presents the offset for each condition for the color report trials (Figure 4a) and the orientation report trials (Figure 4b). For the color report trials, a multiple repeated measures ANOVA showed a significant interaction between the cue type and interference condition (*F*(1, 22) = 5.728, *p* = 0.026, $\eta_{p}^{2}$ = 0.207) and significant main effects for the cue type (*F*(1, 22) = 21.108, *p* < 0.001, $\eta_{p}^{2}$ = 0.490) and the interference condition (*F*(1, 22) = 6.739, *p* = 0.016, $\eta_{p}^{2}$ = 0.234). Follow-up comparisons revealed that the offset was significantly smaller in the valid-cue trials than in the neutral-cue trials under the no-mask condition (*t*(22) = 5.049, *p*_bonf_ *<* 0.001, Cohen's *d* = 1.053, *BF*_10_ = 538.707). However, there was no significant difference in offsets between the valid-cue trials and neutral-cue trials under the mask condition (*t*(22) = 2.004, *p*_bonf_ *=* 0.058, Cohen's *d* = 0.418, *BF*_10_ = 1.184). Additionally, a significant difference in offsets was detected between the mask and no-mask conditions in the valid-cue trials (*t*(22) = 3.250, *p*_bonf_ = 0.004, Cohen's *d* = 0.678, *BF*_10_ = 11.476) but not in the neutral-cue trials (*t*(22) = 0.355, *p*_bonf_ = 0.726, Cohen's *d* = 0.074, *BF*_10_ = 0.232).

For the orientation report trials, a multiple repeated measures ANOVA showed a significant interaction between the cue type and interference condition (*F*(1, 22) = 4.898, *p* = 0.038, $\eta_{p}^{2}$ = 0.182) and significant main effects of the cue type (*F*(1, 22) = 8.096, *p* = 0.009, $\eta_{p}^{2}$ = 0.269) and the interference condition (*F*(1, 22) = 7.483, *p* = 0.012, $\eta_{p}^{2}$ = 0.254). Follow-up comparisons showed that the offset was significantly smaller in the valid-cue trials than in neutral-cue trials under the no-mask condition (*t*(22) = 3.290, *p*_bonf_ = 0.003, Cohen's *d* = 0.686, *BF*_10_ = 12.451), but the offset was comparable in both cue type trials under the mask condition (*t*(22) = 0.117, *p*_bonf_ = 0.908, Cohen's *d* = 0.024, *BF*_10_ = 0.220). A significant difference was detected between the mask condition and no-mask condition only in the valid-cue trials (*t*(22) = 3.330, *p*_bonf_ = 0.003, Cohen's *d* = 0.694, *BF*_10_ = 13.497), but not in the neutral-cue trials (*t*(22) = 0.434, *p*_bonf_ = 0.669, Cohen's *d* = 0.090, *BF*_10_ = 0.238).

#### RBI

The average RBIs are also presented for the color report trials (Figure 4c) and orientation report trials (Figure 4d). For the color report trials, the one sample *t*-test revealed that the RBI in the no-mask condition was significantly larger than 0 (*t*(22) = 5.622, *p*_bonf_ < 0.001, Cohen's *d* = 1.172, *BF*_10_ = 3733.756). However, the RBI in the mask condition did not significantly differ from 0 (*t*(22) = 1.443, *p*_bonf_ = 0.082, Cohen's *d* = 0.301, *BF*_10_ = 0.987). The paired-samples *t*-test revealed that the RBI was significantly smaller in the mask condition than in the no-mask condition (*t*(22) = 2.489, *p*_bonf_ = 0.010, Cohen's *d* = 0.519, *BF*_10_ = 5.262).

For the orientation report trials, the one sample *t*-test revealed that the RBI was significantly larger than 0 in the no-mask condition (*t*(22) = 2.999, *p*_bonf_ = 0.003, Cohen's *d* = 0.625, *BF*_10_ = 13.860), but the RBI in the mask condition did not significantly differ from 0 (*t*(22) = 0.509, *p*_bonf_ = 0.692, Cohen's *d* = 0.106, *BF*_10_ = 0.155). The paired-samples *t*-test revealed that the RBI was significantly smaller in the mask condition than in the no-mask condition (*t*(22) = 2.448, *p*_bonf_ = 0.011, Cohen's *d* = 0.510, *BF*_10_ = 4.886).

### Discussion

Our aim in Experiment 2 was to investigate the effect of perceptual interference on the dimension-based RCB during the stage involving the deployment of attention. We accomplished this using a short cue-and-interference SOA. Our results identified a robust dimension-based RCB under the no-mask condition for both the color report trials and the orientation report trials, in agreement with previous studies on the dimension-based RCB (Hajonides et al., 2020; Heuer & Schubo, 2016; Niklaus et al., 2017; Park et al., 2017; Ye et al., 2016; Ye et al., 2021). More importantly, we found that although the mask did not negatively impact performance in the neutral cue condition, the dimension-based RCB was reduced to complete absence under the mask condition in both report trials. These findings indicate that the dimension-based RCB is sensitive to perceptual interference during the stage involving the deployment of attention, suggesting that when participants use retro-cues to redirect their attention to a specific dimension, they require sustained attention to achieve the dimension-based RCB.

Previous research has established that perceptual interference occurring with a short cue-and-interference SOA (during the stage involving the deployment of attention) can impair the object-based RCB in recall tasks (Hollingworth & Maxcey-Richard, 2013; Rerko et al., 2014; van Moorselaar et al., 2014). However, even though the object-based RCB is damaged, it persists. By contrast, our findings indicate that the dimension-based RCB is completely eliminated by perceptual interference. This suggests that obtaining the dimension-based RCB may require more stringent sustained attention, specifically during the stage involving the deployment of attention, than is required to obtain the object-based RCB. This is reasonable, because participants who are using object-based retro-cues only need to shift their attention to focus on one object, whereas participants who are using dimension-based retro-cues need to shift their attention among all objects.

An important point to note is that, in comparison to Experiment 1, Experiment 2 used a shorter SOA, but it also featured a number of different experimental setups, such as an increased memory load and improved interference. These differences in experimental design and difficulty make any direct comparison of the results of Experiment 2 and Experiment 1 inadvisable. Instead, the results of Experiment 2 should be considered as evidence of the weakening effect of perceptual interference during the stage involving the deployment of attention on the dimension-based RCB.

## Experiment 3

In Experiments 1 and 2, we examined the impact of bottom-up interference from masks on the dimension-based RCB. Experiment 3 was a further investigation of the effect of top-down interruption—specifically, an attention-demanding task at the stage for the maintenance of prioritized (cued) information—on the dimension-based RCB. Previous research on object-based RCB has commonly involved the use of attention-demanding secondary tasks to interrupt attention focus and investigate the effect of an interruption (Hollingworth & Maxcey-Richard, 2013; Janczyk & Berryhill, 2014; Makovski & Pertzov, 2015; Rerko et al., 2014).

In Experiment 3, inspired by the study of Makovski and Pertzov (2015), we chosed an odd-even task as the interruption task and the same cue-and-interruption ISI of 1000 ms as we used in Experiment 1. The interruption task was used between the retro-cue offset and the onset of the test array in the recall task under dual-task conditions. Participants were required to concurrently decide whether the presented number was odd or even while maintaining representations in VWM. Furthermore, we ensured a robust dimension-based RCB by setting the memory load of the recall task at three items.

### Method

#### Participants

We ensured a sufficient sample by using the same reasoning as in Experiment 1 and Experiment 2 to recruit a new sample of 23 participants (19 females and four males; 20.43 ± 0.95 years old, age range 19–22 years; one was left-handed). All participants were college or postgraduate students, with normal or corrected-to-normal vision and no history of neurological issues. They provided written informed consent and received monetary compensation for their participation. Our study received ethical approval from the ethical committee of Sichuan Normal University and followed the guidelines outlined in the Declaration of Helsinki (2008).

#### Materials, apparatus, and procedure

**Figure 5.**
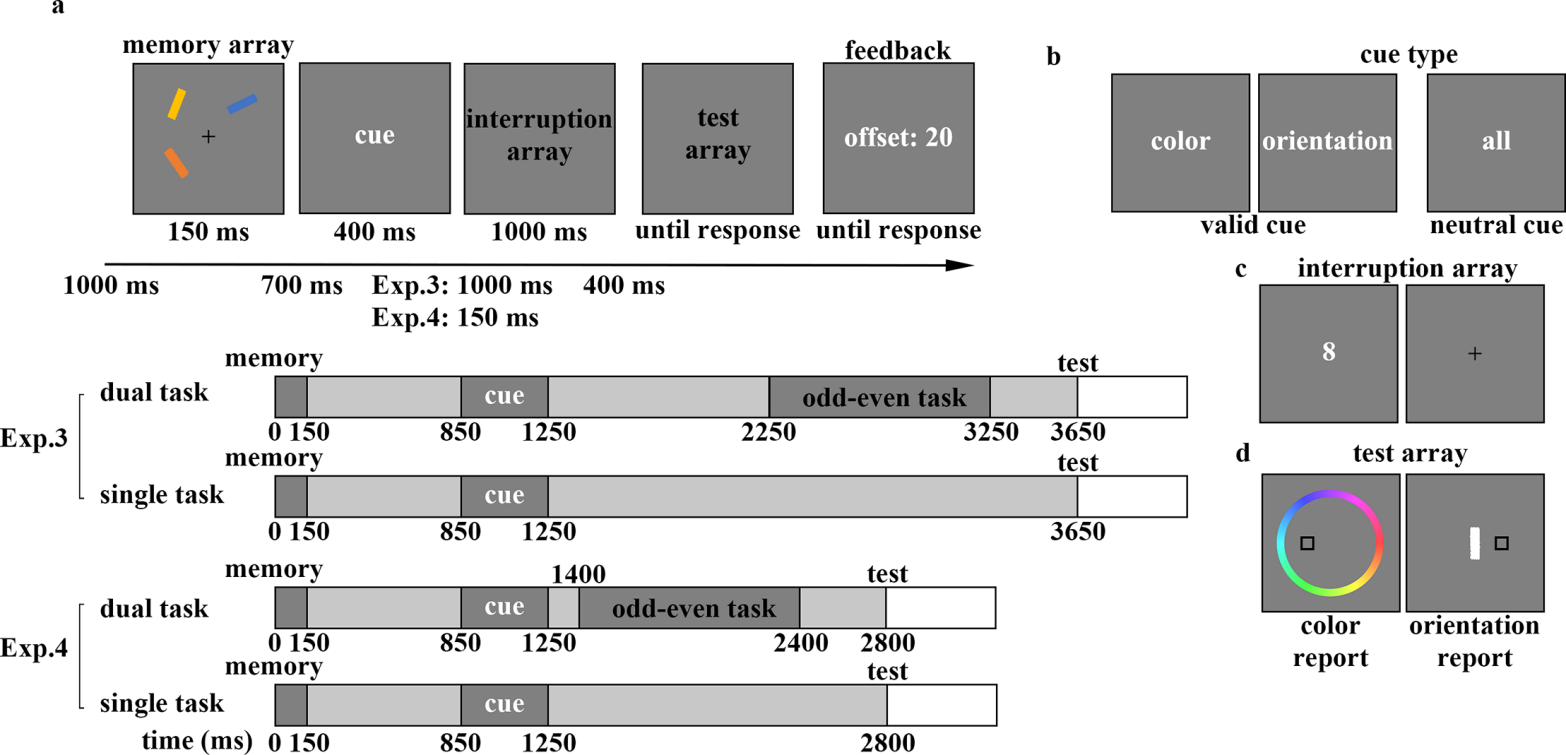
The flowchart and timeline of a recall task in Experiments 3 and 4 (a). Half the trials contained neutral cues showing the word “all” (in Chinese) and the other half displayed valid cues, words implying the to-be-tested dimension (b). In two-thirds of the trials, the participants were asked to conduct a digital odd-even task (c, left), in which an odd or even number existed on the screen with the equal probability, during the interruption array; in the other trials, participants just saw a fixation (c, right) after the cue. When the color was probed, a color wheel appeared as an option (d, left) and when the orientation of target bar was to be reported, a white bar appeared in the center of the screen (d, right).

The apparatus and materials for the recall task in Experiment 3 were the same as those used in Experiment 1. In Experiment 3, we used four single-task conditions (valid cue–single-task–color, neutral cue–single-task–color, valid cue–single-task–orientation, neutral cue–single-task–orientation) and four dual-task conditions (valid cue–dual-task–color, neutral cue–dual-task–color, valid cue–dual-task–orientation, neutral cue–dual-task–orientation). The single-task conditions were similar to the no-mask conditions in Experiment 1. The procedures in Experiment 3 were modified from those in Experiment 1 (shown in Figure 5) with the following changes: (1) The mask condition was replaced by a dual-task condition. The mask in Experiment 1 was replaced by an odd-even task lasting 1000 ms. In the digital judgment task, a random digit (0–9) was presented for 1000 ms, and the participants were asked to respond as quickly and accurately as possible. They had to press “1” when they saw an odd number and press “2” otherwise, within the time limit. In the dual-task trials, an odd or even number appeared in the interruption array with an equal probability (50%) on each trial. (2) The maintenance interval in the single-task trials was increased. The ISI between the cue and the test array in the single task was increased to 2400 ms. (3) The memory load was increased. As in Experiment 2, the total number of items in the memory array was increased to three. (4) The number of trials was adjusted. Specifically, the number of trials in each single-task condition (valid cue–single-task–color, neutral cue–single-task–color, valid cue–single-task–orientation, neutral cue–single-task–orientation) was set to 32, whereas the number of trials in each dual-task condition (valid cue–-dual-task–color, neutral cue–-dual-task–color, valid cue–dual-task–orientation, neutral cue–dual-task–orientation) was set to 64. This resulted in a total of 384 trials, which were fully randomized to minimize experimental bias. The entire experiment lasted approximately 60 minutes and included instructions and at least 16 practice trials before the main task.

#### Data analysis

For the odd-even interruption task, we calculated the average correct rate and the correct reaction time in the whole experiment. Trials with errors in the odd-even task were excluded from subsequent analyses. All subsequent analyses for the recall task, such as offset results and RBI results, were conducted using the same methods described for Experiment 2. In addition, we also report the SD_error_ results in the Supplementary Materials.

### Results

#### Odd-even interruption task

Participants demonstrated a response time of under 1000 ms in 89.22% ± 0.09 of trials, with an average accuracy rate of 0.92 ± 0.07 in their digit judgments. The average response time for correct trials was 761.75 ± 59.64 ms.

#### Recall task

##### Offset

**Figure 6.**
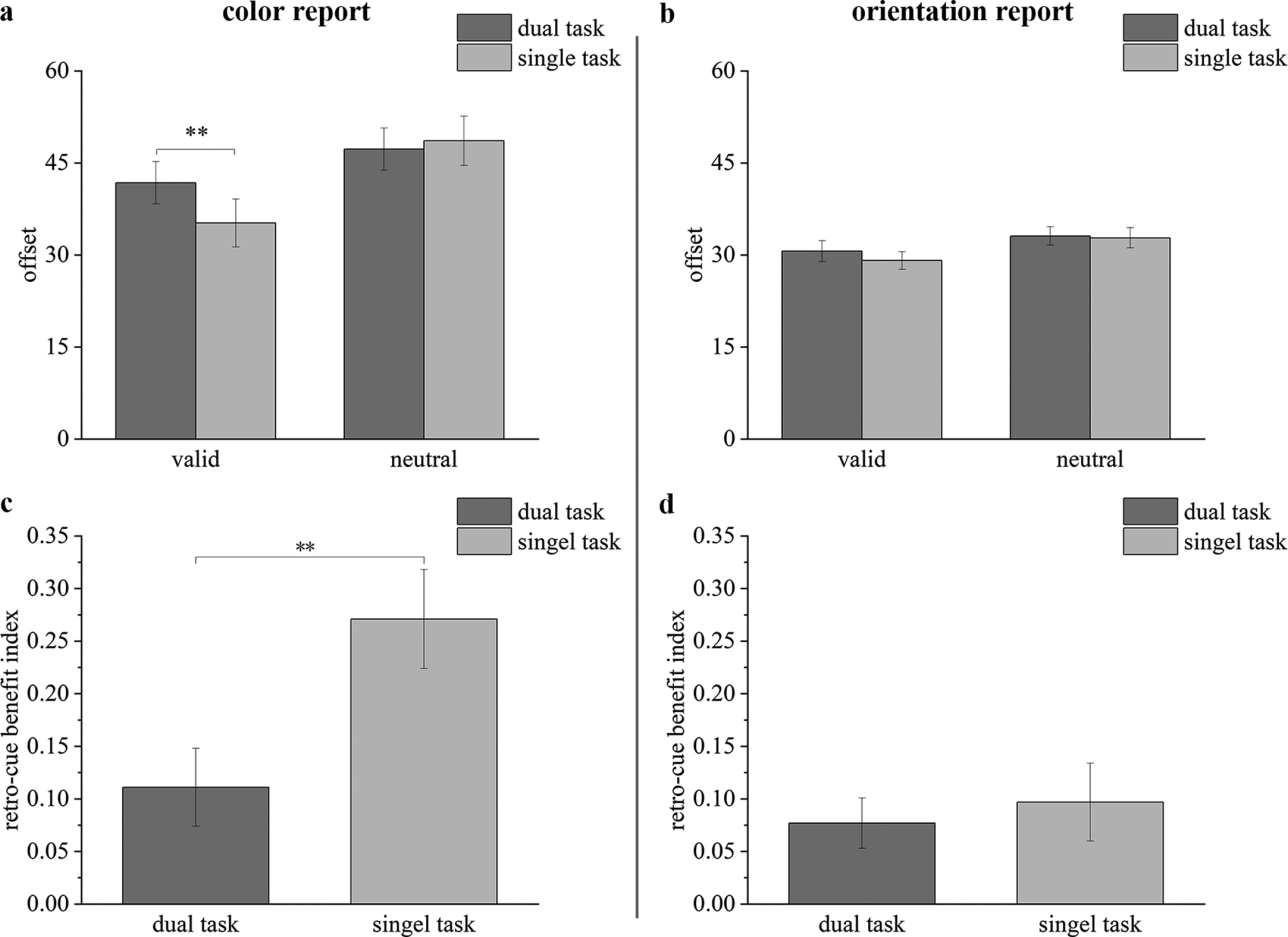
Results of Experiment 3 for the mean offsets (a: color report trials; b: orientation report trials) and RBIs (c: color report trials; d: orientation report trials). The dark gray bars represent results in the dual-task condition; the light gray bars represent results in the single-task condition. ** *p* < 0.01. Error bars reflect within-subject SEMs.

The results of Experiment 3 are shown in Figure 6, which presents the offset for each condition for the color report trials (Figure 6a) and the orientation report trials (Figure 6b). For the color report trials, a multiple repeated measures ANOVA for the offsets with different cue types (neutral cue vs. valid cue) and task conditions (single-task vs. dual-task) as within-subject factors revealed a significant interaction between the cue type and task condition (*F*(1, 22) = 7.700, *p* = 0.011, $\eta_{p}^{2}$ = 0.259) and a significant main effect for the cue type (*F*(1, 22) = 25.554, *p* < 0.001, $\eta_{p}^{2}$ = 0.537) but no significant main effect was evident for the task condition (*F*(1, 22) = 2.439, *p* = 0.133, $\eta_{p}^{2}$ = 0.100). Follow-up comparisons revealed that the offset was significantly smaller in valid-cue trials than in neutral-cue trials under both the dual-task condition (*t*(22) = 2.529, *p*_bonf_ = 0.019, Cohen's *d* = 0.527, *BF*_10_ = 2.868) and the single-task condition (*t*(22) = 5.316, *p*_bonf_ < 0.001, Cohen's *d* = 1.109, *BF*_10_ = 964.473). A significant difference was also found between the dual-task condition and single-task condition in the valid-cue trials (*t*(22) = 3.400, *p*_bonf_ = 0.003, Cohen's *d* = 0.709, *BF*_10_ = 15.585), but not in the neutral-cue trials (*t*(22) = 0.557, *p*_bonf_ = 0.583, Cohen's *d* = 0.116, *BF*_10_ = 0.252).

For the orientation report trials, a multiple repeated measures ANOVA showed no significant interaction between the cue type and task condition (*F*(1, 22) = 1.255, *p* = 0.275, $\eta_{p}^{2}$ = 0.054) and no significant main effect for the task condition (*F*(1, 22) = 1.290, *p* = 0.268, $\eta_{p}^{2}$ = 0.055), but a significant main effect was found for the cue type (*F*(1, 22) = 15.317, *p* < 0.001, $\eta_{p}^{2}$ = 0.410). Follow-up comparisons showed that the offset was significantly smaller in valid-cue trials than in neutral-cue trials under both the dual-task condition (*t*(22) = 3.097, *p*_bonf_ = 0.005, Cohen's *d* = 0.646, *BF*_10_ = 8.438) and the single-task condition (*t*(22) = 3.344, *p*_bonf_ = 0.003, Cohen's *d* = 0.697, *BF*_10_ = 13.907). Planned comparisons demonstrated no significant difference between the dual-task condition and single-task condition in the valid-cue trials (*t*(22) = 1.556, *p*_bonf_ = 0.134, Cohen's *d* = 0.324, *BF*_10_ = 0.626) or in the neutral-cue trials (*t*(22) = 0.313, *p*_bonf_ = 0.758, Cohen's *d* = 0.065, *BF*_10_ = 0.229).

##### RBI

The average RBIs are also illustrated in the color report trials (Figure 6c) and the orientation report trials (Figure 6d).

For the color report trials, the one sample *t*-test revealed that the RBI was significantly larger than 0 both in the dual-task condition (*t*(22) = 2.965, *p*_bonf_ = 0.004, Cohen's d = 0.618, *BF*_10_ = 12.948) and in the single-task condition (*t*(22) = 5.758, *p*_bonf_ < 0.001, Cohen's *d* = 1.201, *BF*_10_ = 4999.703). The paired-samples *t*-test showed that the RBI was significantly smaller in dual-task condition than in single-task condition (*t*(22) = 3.185, *p*_bonf_ = 0.002, Cohen's *d* = 0.664, *BF*_10_ = 20.065).

For the orientation report trials, the one sample *t*-test showed that the RBI was significantly larger than 0 both in the dual-task condition (*t*(22) = 3.192, *p*_bonf_ = 0.002, Cohen's *d* = 0.666, *BF*_10_ = 20.361) and in the single-task condition (*t*(22) = 2.634, *p*_bonf_ = 0.008, Cohen's *d* = 0.549, *BF*_10_ = 6.877). The paired-samples *t*-test revealed no significant difference between the dual-task condition and single-task condition (*t*(22) = 0.543, *p*_bonf_ = 0.296, Cohen's *d* = 0.113, *BF*_10_ = 0.348).

### Discussion

In Experiment 3, the dimension-based RCB was significantly impaired by the presence of the interruption task in the color report trials. However, no significant interruption effect was evident on the dimension-based RCB in the orientation report trials, despite the RBI being numerically smaller in the dual-task condition than in the single-task condition. This indicates that the maintenance of attention to the orientation dimension may require less effort to resist top-down interruption. These findings suggest that top-down interruption occurring after the prioritization of cued dimension-based representation impairs the dimension-based RCB after attention is directed to the color dimension. At least, the selective maintenance of color information demands sustained attention. It is worth noting that although the secondary task weakened the dimension-based RCB, it did not disappear it completely. This may be due to the relatively low difficulty level of the odd-even interruption task and the fact that it did not fully engage the sustained attention of the participants.

Despite the well-documented VWM improvement effect of valid retro-cues, irrespective of the presence or absence of a post-cue secondary task, our findings reveal that the interruption did not affect the dimension-based RCB of orientation. This contrasts with the results of Makovski and Pertzov (2015), who reported an increase in object-based RCB of orientation in dual-task trials compared to single-task trials. The discrepancy can be attributed to different interruption effects, particularly on neutral-cue trials. We observed no interruption effect on either valid-cue or neutral-cue trials. In contrast, Makovski and Pertzov (2015) reported that the odd-even task impaired VWM performance for both valid-cued and neutral-cued orientation, and, compared to the no-interruption condition, the boosted effect of retro-cue followed by an interruption task resulted from worse performance for neutral-cue trials rather than better performance for valid-cue trials. That is, in the study by Makovski and Pertzov (2015), the interruption task resulted in impaired VWM representation, and RCB can reduce this damage. However, in our Experiment 3, we found that the interruption task did not significantly impair VWM representation. These findings suggest that different attention mechanisms may underlie the processing of single-dimension and multidimension stimuli. Despite similar VWM capacities and contralateral delay activity amplitudes between the two categories of stimuli, participants consistently report higher task difficulty and require more effort to remember the same number of targets with multi-dimension stimuli (Luria & Vogel, 2011; Vogel et al., 2001; Woodman & Vogel, 2008). In Experiment 3, participants may have needed to adopt a more effective strategy to store representations in the subjectively more challenging two-dimension VWM task. As a result, memory array may have been represented in a passive state (Stokes, 2015) in the neutral-cue trials of Experiment 3 and these representations were robust and suffered little memory loss from the interruption by the odd-even task (See the General Discussion section for further elaboration). However, in the interruption condition of the study by Makovski and Pertzov (2015), the orientation representations in an active state were vulnerable to swap errors because of the absence of sustained attention, which is crucial for reinforcing location information. In the valid-cue trials, when a certain item is prioritized, the orientation-location correspondence may be protected. Consequently, the impairment degree of interruption shows a discrepancy between valid-cue and neutral-cue trials, leading to an increase of object-based RCB under the interruption condition.

In Experiment 3, the total number of trials in the dual-task conditions was double that of the single-task conditions, and all trials were fully randomized. This was done to ensure that the participants were aware that interruption tasks would occur frequently, and that they would need to be prepared to allocate attention to the odd-even task after the retro-cue disappeared in each trial. Despite this, we still observed a robust dimension-based RCB under the single-task (no-interruption) conditions for both the color report trials and the orientation report trials, similar to the results of Experiment 2. This suggests that our trial design was effective and that the dimension-based RCB was not influenced by the attentional preparation state of the participants. Furthermore, this trial design also ensured that we had a sufficient number of valid trials for data analysis in the dual-task conditions after excluding the incorrect trials in the interruption task.

Notably, unlike Experiment 1 and Experiment 2, which used masks as the perceptual interference, Experiment 3 used an attention-demanding secondary task as a cognitive interruption. The perceptual interference occupies attention resources in a bottom-up manner, while cognitive interruption occupies attention resources in a top-down manner. These differences in experimental design make direct comparison of the results of Experiment 3 to those of Experiment 1 and Experiment 2 inadvisable. Instead, the results of Experiment 3 should be considered as evidence for the negative impact of cognitive interruption on the dimension-based RCB, particularly in the color report trials.

## Experiment 4

In Experiment 3, we investigated the effects of top-down interruption on dimension-based RCB when participants were given sufficient time to select relevant information in VWM prior to the interruption during the maintenance stage. To further explore the effect of cognitive interruption on dimension-based RCB, we conducted Experiment 4 to examine how top-down interruption would impact the dimension-based RCB during the stage involving the deployment of attention. We implemented an odd-even task after the retro-cue display in the dual-task (interruption) condition with a short cue-and-interruption SOA of 550 ms. This SOA was composed of a retro-cue presented for 400 ms, which was the same display time as that in Experiment 3, and a 150 ms cue-and-interruption ISI, which was the same as that in Experiment 2.

### Method

#### Participants

We ensured a sufficient sample by using the same reasoning as the above three experiments to recruit 26 new participants, two of which were refused from the following data analyses because of accuracy rates under 0.75 in the secondary odd-even task, leaving a sample of 24 participants (19 females and 5 males; 19.75 ± 1.51 years old, age range 18–25 years; right-handed). All participants were college or postgraduate students, with normal or corrected-to-normal vision and no history of neurological issues. They provided written informed consent and received monetary compensation for their participation. Our study received ethical approval from the ethical committee of Sichuan Normal University and followed the guidelines outlined in the Declaration of Helsinki (2008).

#### Materials, apparatus and procedure

The apparatus, materials and procedure for the recall task in Experiment 4 were the same as those used in Experiment 3 (shown in Figure 5), with the exception that the post-cue interval in dual-task trials was shortened to 150 ms, forming a 550 ms cue-and-interruption SOA and correspondingly, the ISI between cue and test displays in single-task trials was adjusted to 1550 ms.

#### Data analysis

The data analysis for Experiment 4 was conducted as described for Experiment 3.

### Results

#### Odd-even interruption task

Participants demonstrated a response time of under 1000 ms and over 100 ms in 94.94% ± 0.03% of trials, with an average accuracy rate of 0.92 ± 0.05 in their digit judgments. The average response time for correct trials was 698.77 ± 45.22 ms.

#### Recall task

##### Offset

**Figure 7.**
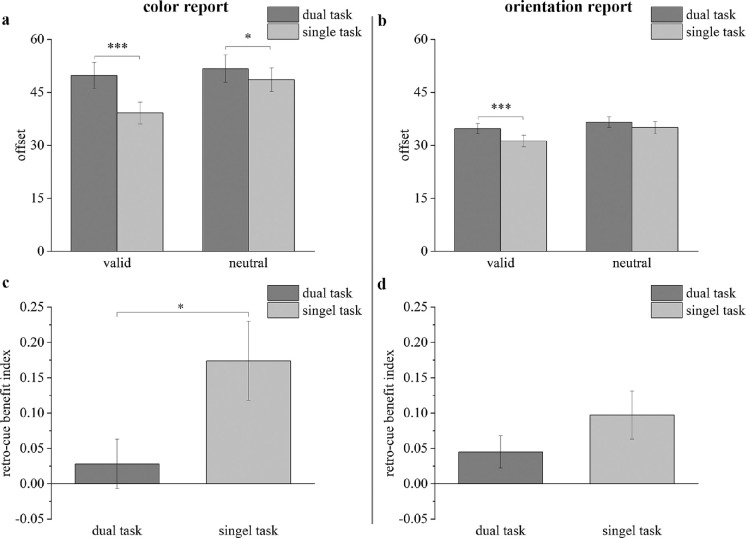
Results of Experiment 4 for the mean offsets (a: color report trials; b: orientation report trials) and RBIs (c: color report trials; d: orientation report trials). The dark gray bars represent results in the dual-task condition; the light gray bars represent results in the single-task condition. * *p* < 0.05, *** *p* < 0.001. Error bars reflect within-subject SEMs.

The results of Experiment 4 are shown in Figure 7 which presents the offset for each condition for the color report trials (Figure 7a) and the orientation report trials (Figure 7b). For the color report trials, a multiple repeated measures ANOVA with different cue types (neutral cue vs. valid cue) and task conditions (single-task vs. dual-task) revealed a significant interaction between the cue type and task condition (*F*(1, 23) = 8,626, *p* = 0.007, $\eta_{p}^{2}$ = 0.273). We also found a significant main effect both for the cue type (*F*(1, 23) = 9.392, *p* = 0.005, $\eta_{p}^{2}$ = 0.290) and for the task condition (*F*(1, 23) = 21.685, *p* < 0.001, $\eta_{p}^{2}$ = 0.485). Follow-up comparisons revealed that the offset was significantly smaller in valid-cue trials than in neutral-cue trials under the single-task condition (*t*(23) = 3.472, *p*_bonf_ = 0.002, Cohen's *d* = 0.709, *BF*_10_ = 18.633), but there was no significant difference in offsets between valid-cue trials and neutral-cue trials under the dual-task condition (*t*(23) = 1.176, *p*_bonf_ = 0.252, Cohen's *d* = 0.240, *BF*_10_ = 0.379). A significant difference was also found between the single-task condition and dual-task condition in both the valid-cue trials (*t*(23) = 4.594, *p*_bonf_ < 0.001, Cohen's *d* = 0.938, *BF*_10_ = 217.036) and the neutral-cue trials (*t*(23) = 2.106, *p*_bonf_ = 0.046, Cohen's *d* = 0.430, *BF*_10_ = 1.381).

For the orientation report trials, a multiple repeated measures ANOVA showed no significant interaction between the cue type and task condition (*F*(1, 23) = 1.461, *p* = 0.239, $\eta_{p}^{2}$ = 0.06) but a significant main effect both for the cue type (*F*(1, 23) = 19.388, *p* < 0.001, $\eta_{p}^{2}$ = 0.457) and for the task condition (*F*(1, 23) = 13.006, *p* = 0.001, $\eta_{p}^{2}$ = 0.361). Planned comparisons showed that the offset was significantly smaller in valid-cue trials than in neutral-cue trials under both the dual-task condition (*t*(23) = 2.217, *p*_bonf_ = 0.037, Cohen's *d* = 0.452, *BF*_10_ = 1.659) and the single-task condition (*t*(23) = 3.091, *p*_bonf_ = 0.005, Cohen's *d* = 0.631, *BF*_10_ = 8.484). There was a significant difference between the single-task condition and dual-task condition in the valid-cue trials (*t*(23) = 5.270, *p*_bonf_ < 0.001, Cohen's *d* = 1.076, *BF*_10_ = 983.180), but the significant difference between the task conditions was not detected in the neutral-cue trials (*t*(23) = 1.116, *p*_bonf_ = 0.276, Cohen's *d* = 0.228, *BF*_10_ = 0.374).

##### RBI

The average RBIs are also illustrated in the color report trials (Figure 7c) and the orientation report trials (Figure 7d). For the color report trials, the one sample *t*-test revealed that the RBI was significantly larger than 0 in the single-task trials (*t*(23) = 3.093, *p*_bonf_ = 0.005, Cohen's *d* = 0.631, *BF*_10_ = 8.510), whereas the RBI in the dual-task condition did not significantly differ from 0 (*t*(23) = 0.786, *p*_bonf_ = 0.440, Cohen's *d* = 0.160, *BF*_10_ = 0.284). The paired-samples *t*-test showed that the RBI was significantly smaller in dual-task condition than in single-task condition (*t*(23) = 2.685, *p*_bonf_ = 0.013, Cohen's *d* = 0.548, *BF*_10_ = 3.836).

For the orientation report trials, the one sample *t*-test showed that the RBI was significantly larger than 0 in the single-task trials (*t*(23) = 2.827, *p*_bonf_ = 0.010, Cohen's *d* = 0.577, *BF*_10_ = 5.032), but the RBI in the dual-task condition did not significantly differ from 0 (*t*(23) = 1.979, *p*_bonf_ = 0.060, Cohen's *d* = 0.404, *BF*_10_ = 1.128). The paired-samples *t*-test revealed no significant difference between the single-task condition and dual-task condition (*t*(23) = 1.183, *p*_bonf_ = 0.249, Cohen's *d* = 0.241, *BF*_10_ = 0.400).

### Discussion

In Experiment 4, we observed a significant impairment of the dimension-based RCB in color report trials when a task requiring top-down attention was introduced at a short cue-and-interference SOA. This finding indicates that sustained attention to color information is essential for maintaining the dimension-based RCB when attentional deployment is directed by a valid retro-cue. Our results are consistent with those of our Experiment 2 and some previous studies on the object-based RCB (Pinto et al., 2013; van Moorselaar et al., 2014), suggesting that VWM begins to allocate attention and other resources to task-relevant information after the appearance of a valid retro-cue. During the stage involving the deployment of attention, attentional focus on the task-relevant information is crucial, and top-down interruption can hinder target prioritization, resulting in the attenuation and even absence of RCB in dual-task trials.

For the orientation report results, it should be noted that a significant reduction in the RBI of orientation was not observed in the dual-task condition compared to the single-task condition. However, the multiple repeated measures ANOVA conducted on the SD_error_ of orientation reports revealed a significant interaction effect. Follow-up comparisons for the SD_error_ showed a similar pattern of results to those obtained for the offset, except that no RCB was detected in the dual-task condition (see the Supplementary Materials for more details on the SD_error_ results). Moreover, both the offset and SD_error_ results indicate that VWM performance was worse in dual-task condition than in single-task condition only in the valid-cue trials. These findings suggest that cognitive interruption at a short cue-and-interruption SOA may have a differential impact on valid-cued and neutral-cued orientation information in VWM. These findings are consistent with those of Experiment 2, which suggest that sustained attention is necessary for the biased deployment of attention following the appearance of a valid retro-cue.

In addition, the results of SD_error_ show that no significant difference of color VWM exists between single-task and dual-task conditions in the neutral-cue trials, similar to the offset and SD_error_ results of orientation report trials. These findings suggest that the unbiased maintenance of VWM representations can effectively resist attention-demanding interruptions. One possible explanation for this phenomenon is that VWM information may be represented in the passive state of VWM because of the long SOA (i.e., 1400 ms) between the memory array and interruption tasks. Our previous experiments have shown that when the interval between the initial memory information and subsequent presentation stimuli is long enough (e.g., more than 800 ms), the initial memory information can be transferred to the passive state (Li, Zhang, Liang, Ye, & Liu, 2020). Thus these representations stored in the passive state of VWM were not impaired by the interruption tasks, unlike the representations stored in the active state of VWM (for more details about storage states, see the General Discussion section).

It is worth noting that a better average offset of color reports was observed in neutral-cue trials with a single task than with dual-tasks in the follow-up comparison. However, the Bayes factor analysis for this difference (the interruption task impaired VWM performance in the neutral-cue condition) was close to 1 (*BF*_10_ = 1.381), which suggests anecdotal evidence for the alternative hypothesis. Therefore we refrained from drawing a strong conclusion based on the offset results in the neutral-cue condition of the color report trials and did not further discuss how interruption tasks impaired VWM representations in this specific case.

The results of Experiment 3 indicated that VWM performance remained stable regardless of interruptions to sustained attention during the maintenance process of prioritized information, thereby resulting in a consistent dimension-based RCB. However, the results of Experiment 4 suggest that the effect of interruption is only observable in valid-cue trials. Furthermore, no apparent RBI is observed in dual-task trials, indicating that the interruption at the stage involving the deployment of attention eliminates the RCB of orientation. A crucial difference between Experiment 4 and Experiment 3 is the timing of when the interruption occurs. It is possible that during the stage involving the deployment of attention, the selection of the cued representation relies on sustained attention, and, as a result, the interruption impairs prioritization. After attentional deployment, the maintenance of prioritized information requires less attention, and therefore, the removal of attention results in less loss of RCB.

In summary, the results of Experiment 4 suggest that the introduction of an interruption task can abolish the dimension-based RCB when participants are in the process of reallocating attention using the retro-cue.

## General discussion

In this study, we examined whether the dimension-based RCB requires sustained attention. In four experiments, interferences or interruptions were introduced in the interval between the retro-cue and the test array to distract attention. Through these experiments, we evaluated the impact of interference or interruption on the dimension-based RCB at a long cue-and-interference/interruption SOA (1400 ms in Experiment 1 and Experiment 3) or a short cue-and-interference/interruption SOA (400 ms in Experiment 2 and 550 ms in Experiment 4). Our results indicated that the dimension-based RCB was reduced by both perceptual interference (masks, Experiment 1 and Experiment 2) and cognitive interruption (odd-even task, Experiment 3 and Experiment 4), implying that sustained attention is important for the prioritization of the cued dimension in VWM representations and decides the effectiveness of the dimension-based RCB.

Our findings also suggest that impaired sustained attention can weaken the dimension-based RCB, regardless of when the interference or interruption occurs. This finding contrasts with previous studies on object-based RCB (Hollingworth & Maxcey-Richard, 2013; Rerko et al., 2014; van Moorselaar et al., 2014), which have shown that secondary interruption tasks only impair the RCB when the cue-and-interruption SOA is short. Considering this pattern and the account put forth by Janczyk and Berryhill (2014), we propose that the VWM process of selected representation after the appearance of valid retro-cue may be divided into two steps: the deployment of attention and the maintenance of prioritized information. When interference or interruption occurs shortly after a valid retro-cue, impaired sustained attention during the deployment of attention disrupts participants' ability to effectively use a retro-cue to prioritize a specific dimension, thereby reducing the RCB. Interestingly, our findings indicate that even when the stage involving the deployment of attention was completed, interference or interruption during the stage for the maintenance of prioritized information was still capable of reducing the dimension-based RCB, while leaving the object-based RCB unaffected. This effect discrepancy between dimension-based RCB and object-based RCB may arise from differences in the amount of selected representation targeted by the dimension-based retro-cue (i.e., multiple one-feature representations) versus the object-based retro-cue (i.e., one double-feature representation). After using the dimension-based retro-cue, the participants still need to use global attention to prioritize the information of each item. At this time, the intrusion of stimulus-driven interference or top-down interruption on sustained attention will hinder this global attention. Consequently, the prioritization effect for a specific dimension of all items was weakened. To recap, even if the dimension-based RCB and the object-based RCB may share some overlapping attentional mechanisms, some differences remain in the requirement for sustained attention for these benefits.

In our study, we evaluated the effect of two routes to disrupt sustained attention on the dimension-based RCB: a stimulus-driven perceptual interference of irrelevant visual information (i.e., masks) and a top-down cognitive interruption of attention-demanding tasks (i.e., odd-even task). The interference and interruption can have different effects on cognitive processes (Katsuki & Constantinidis, 2014) and different functions in neurophysiology (Bowling, Friston, & Hopfinger, 2020; Long & Kuhl, 2018). For example, VWM representation was robust to the presence of letters (stimulus-driven visual information) but was negatively impacted by a letter change detection task (top-down cognitive task) (Wang, Theeuwes, & Olivers, 2018). However, our research findings suggest that both interference and interruption impaired the dimension-based RCB. Therefore we suggest that the decrease in the dimension-based RCB depends solely on whether sustained attention is disrupted, rather than on the specific mechanism by which stimuli cause the disruption.

Our prior study on the dimension-based RCB proposes that the dimension-based RCB likely arises due to a combination of two mechanisms: a strengthening of information related to the cued dimension (strengthening hypothesis) and a removal of information related to the non-cued dimension (removal hypothesis) (Ye et al., 2016). The strengthening hypothesis posits that the RCB is caused by an elevated status of cued dimension information, while the removal explanation suggests that it is due to a change in the status of non-cued representations. Our findings showed that the dimension-based RCB requires sustained attention to maintain the cued dimension information in a priority state, thereby supporting the strengthening hypothesis. However, our results also indicated that, even in the presence of interference or interruption in the maintenance of prioritized dimension, the dimension-based RCB was not entirely eliminated due to the impairment of sustained attention. This suggests that sustained attention is not the sole cause of the dimension-based RCB.

The removal explanation posits that even if the cued dimension information remains unchanged, removing the non-cued dimension information from the capacity-limited VWM reduces recall competition and interference, thereby improving the recall of the cued dimension information. This process does not require sustained attention; thus an impairment of sustained attention does not affect the removal mechanism's effect on the dimension-based RCB. This can explain the residual dimension-based RCB even after sustained attention is impaired. Therefore our findings are consistent with our original expectation that the dimension-based RCB is achieved through a combination of strengthening and removal mechanisms. Future research can use methods similar to previous studies on the object-based RCB (Rerko & Oberauer, 2013) to further test the contribution of the removal mechanism to the dimension-based RCB through multiple retro-cues.

In our four experiments, we found little evidence that interference or interruption had a negative effect on VWM performance under neutral cue conditions. These results indicate that the interference or interruption in our study mainly impacted participants' sustained attention, whereas it did not directly impair VWM representations. This may reflect the lengthy SOA between the memory array and interference/interruption (2250 ms for Experiment 1; 1250 ms for Experiment 2; 2250 ms for Experiment 3; 1400 ms for Experiment 4) would have allowed participants adequate time to stable VWM representations. According to recent research, VWM information has multiple storage states (Lorenc, Sreenivasan, Nee, Vandenbroucke, & D'Esposito, 2018). Visual information can be represented in an active or passive state, with task relevance impacting its state. Some studies have shown that neurophysiological recordings demonstrate a “ramp-up” of activity during the delay period, when content-specific activity decreases during maintenance, but increases prior to testing (Stokes, 2015). Our recent study showed that information stored in the passive state was robust and suffered little memory loss during latent maintenance (Zhang et al., 2022). In the present study, the cued dimension was highly relevant to the task goal, because of the use of 100% valid cues, and this may have been represented in an active state, supported by a burst of spikes to refresh corresponding synaptic representations (Miller, Lundqvist, & Bastos, 2018). This active representation may have been susceptible to novel interference or interruption also in an active state, at least during encoding. However, VWM representations in the neutral cue conditions were likely stored in a passive state, protected from the impact of interference or interruption. Thus, the information under neutral cue conditions in our study was able to resist disruption.

Different visual features, such as color, orientation, and spatial frequency, are known to be processed by distinct brain modules (Conway, 2009; Paik & Ringach, 2011). The differing result patterns between color and orientation report trials in Experiments 1 and 3 may reflect differences in attention allocation toward these two features (Fougnie & Alvarez, 2011; Wang, Cao, Theeuwes, Olivers, & Wang, 2017). Differences are also evident in the difficulty of retaining color and orientation information, with orientation information occupying more bandwidth and taking more time to consolidate into VWM compared to color information (Hao et al., 2018; Miller et al., 2014). Color and orientation are processed by separate neural populations (Hubel & Wiesel, 1968; Livingstone & Hubel, 1988) and perceptions of these features are largely independent. Color is considered a higher-level visual property and has a specialized population of neurons, while orientation is closely linked to retinal coordinates and no evidence exists for a dedicated area in the visual cortex that would process this dimension. Attention toward the color dimension will also spread to non-focus objects (Niklaus et al., 2017). Color and orientation information of a multi-dimensional object can be stored separately in VWM (Wang et al., 2017). Thus the mechanisms corresponding to internal attention may vary when retro-cues indicate different dimensions, leading to small differences in the effect of interference or interruption on dimension-based report between color and orientation trials. Further research is needed to explore whether the interference/interruption effect on the dimension-based RCB can be extended to other information dimensions (e.g., spatial frequency or shape).

## Conclusion

The current study adds to the growing evidence that sustained attention and improvement in VWM performance are intertwined. Our results support the idea that interference or interruption can negatively impact the dimension-based RCB in a VWM task and that sustained attention is crucial for the dimension-based RCB. The effect of interference or interruption can be understood through a combination of the strengthening and removal hypotheses. Our study sheds new light on the mechanisms of the dimension-based RCB and its differences from the previously reported object-based RCB (Hollingworth & Maxcey-Richard, 2013; Rerko et al., 2014). Future research should use neuroimaging methods to investigate the similarities and differences in the cognitive and neural mechanisms underlying these two forms of the RCB.

## Supplementary Material

Supplement 1
