# Supplementary material for: Sustained attention required for effective dimension-based retro-cue benefit in visual working memory
Source: J Vis. 2023 May 16;23(5):13. doi: 10.1167/jov.23.5.13 (PMC10198287; doi:10.1167/jov.23.5.13)
Supplement: Supplement 1 [file jovi-23-5-13_s001.pdf]

Supplementary Materials

Sustained Attention Required for Effective Dimension-Based Retro-Cue Benefit in Visual Working Memory

Ruyi Liu[1+], Lijing Guo[1,2+], Hong-jin Sun[3], Tiina Parviainen[2,4], Zifang Zhou[1], Yuxin Cheng[1], Qiang Liu[1*], Chaoxiong Ye[1,2,3,5*]

[1] Institute of Brain and Psychological Sciences, Sichuan Normal University, Chengdu, China;
[2] Department of Psychology, University of Jyvaskyla, Jyväskylä, Finland;
[3] Department of Psychology, Neuroscience and Behaviour, McMaster University, Hamilton, Canada;
[4] Centre for Interdisciplinary Brain Research, University of Jyväskylä, Jyväskylä, Finland
[5] Faculty of Social Sciences, Tampere University, Tampere, Finland.

Running Title: dimension-based RCB requires sustained attention

+ Ruyi Liu and Lijing Guo contributed equally to this work and should be considered as co-first authors.

*Correspondence should be addressed to Qiang Liu, Institute of Brain and Psychological Sciences, Sichuan Normal University, Chengdu, 610068, China. E-mail: lq780614@163.com; or Chaoxiong Ye, Institute of Brain and Psychological Sciences, Sichuan Normal University, Chengdu, 610068, China. E-mail: cxye1988@163.com.

Pilot experiment

Previous research has investigated the interference or interruption effect on object-based retro-cue benefit (RCB) in the change detection task (Hollingworth & Maxcey-Richard, 2013; Janczyk & Berryhill, 2014; Makovsik & Jiang, 2007; Rerko et al., 2014). In our pilot experiment, we asked participants to perform a change detection task while manipulating the presence of perceptual interference (masks) after dimension-based retro-cues. The purpose of these masks was to distract sustained attention on the cued dimension information in VWM. Our pilot experiment consisted of the same four conditions used in Experiment 1 in the main text: valid cue–mask condition, valid cue–no mask condition, neutral cue–mask condition, and neutral cue–no mask condition.

Method

Participants

Based on the previous study by Heuer & Schubö (2017) on dimension-based retro-cues with a change detection task, we predicted a similar effect size (η_p^2 = 0.26) for our experimental design. A power analysis (G*Power 3.1.9.2; Faul et al., 2007) indicated that 19 participants were needed to achieve 95% power at an alpha level of 0.05. We ensured an adequate sample size by recruiting 24 participants for SExperiment 1, following the study by van Moorselaar et al (2015) on the perceptual interference effect on object-based RCB (n = 24). These participants were college or postgraduate students (15 females and 9 males; 19.92 ± 1.35 years old; age range 18–23 years; right-handed), who reported having normal or corrected-to-normal vision and no history of neurological problems. They provided written informed consent and received monetary compensation for their participation. Our study was approved by the ethical committee of Sichuan Normal University. All the study's procedures complied with the Declaration of Helsinki (2008).

Materials and apparatus

The memory stimuli consisted of colored arrows with different randomly selected colors and orientations. There were eight colors (orange: 249, 166, 10; pinkish-purple: 221, 160, 220; magenta: 255, 20, 148; green: 128, 255, 0; dark yellow: 160, 82, 46; dark blue: 2, 4, 148; sky blue: 0, 191 ,254; dark green: 59, 98, 96) and eight orientations (15°, 60°, 105°, 150°, 195°, 240°, 285°, and 330°) independently from which the color and orientation of each memory stimulus were randomly selected. Each arrow was 1.2° in length and 0.6° in height, and the color and orientation in a trial were always different. The masks were generated from 18 patterns (0.6° × 0.6°) consisting of eight intertwining arrows with randomly produced combinations of colors and orientations.

The retro-cues were presented as Chinese words and were either "color" or "orientation" for the valid cue, indicating which dimension would be tested, or "all" for the neutral cue, indicating that one of the dimensions would be tested.

The experiment was conducted in a softly lit, soundproof room using 19-inch screens (1280 × 768) with a gray (RGB: 128,128,128) background, and participants were seated approximately 60 cm away from the screen.

Procedure

The experimental procedure is detailed in SFigure 1. Each trial started with a central fixation point (a black cross) appearing on the screen for 1,000 ms, and the participants were instructed to maintain their eye gazing on it throughout the experiment. Two memory stimuli were then presented, located 1.5° to the left or right of the fixation. The memory array was displayed for 150 ms and the participants were asked to remember the color and orientation of the two arrows. A 700 ms blank was followed by the retro-cue, which replaced the fixation cross and was presented for 400 ms. In half the trials, a 100% valid cue appeared (valid cue condition), and in the other half, a neutral cue was presented (neutral cue condition). In the mask condition trials, the cue was sequentially followed by a 1,000 ms blank and the 100 ms mask array, which served to introduce irrelevant information and distract sustained attention

from the cued dimension information. In the other half of the trials, the black cross was displayed instead (no-mask condition). After a 400 ms blank, the test array was presented for 2,500 ms. For the test array, in 50% of the trials, test array was a colored circle (diameter = 0.6°) without any orientation information, and it appeared at the same location as the targeted item in the memory array. Participants were asked to determine whether the color of the test item had changed compared to the target item in the memory array. In the other 50% of the trials, test array was a white arrow (0.6° × 0.6°) without any color information, and participants were asked to determine whether the orientation of the test item had changed. Participants responded by pressing "F" on the keyboard to indicate "same" and "J" to indicate "different". Accuracy was emphasized over response speed, and the test array disappeared upon response. The participants received feedback on their performance during a practice phase, but no feedback was provided during the formal experiment.

The task consisted of 320 trials divided equally into two cue types (neutral cue or valid cue), each of which included 80 trials with masks and 80 trials without masks. Trials with color report and with orientation report were balanced. The trials were fully randomized across the eight conditions (valid cue–mask–color, valid cue–no mask–color, neutral cue–mask–color, neutral cue–no mask–color, valid cue–mask–orientation, valid cue–no mask–orientation, neutral cue–mask–orientation, and neutral cue–no mask–orientation). Each participant completed the one hour task after practicing at least 24 trials, with a short break provided after every 80 trials.

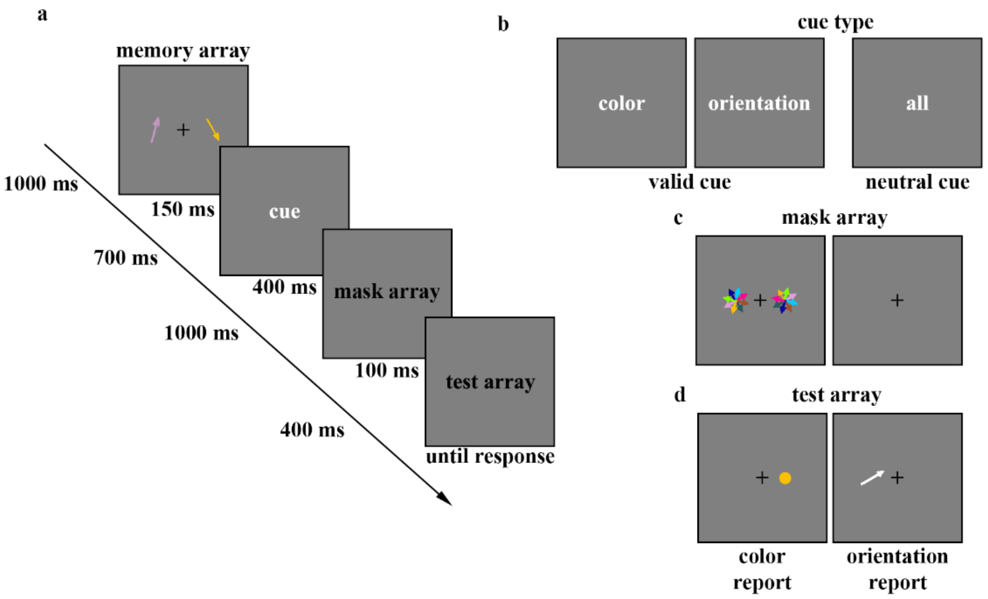

SFigure 1: The flowchart of a change detection task in the pilot experiment (a). Half the trials contained neutral cues showing the word "all" (in Chinese) and the other half displayed valid cues, words implying the to-be-tested dimension (b). In the mask array, we provided two masks on the site of the memoranda (c, left) or nothing but a fixation (c, right) with equal probability. When the color was probed, a colored circle appeared on the site directing to the tested arrow (d, left) and when the orientation was to be reported, a white arrow appeared on the corresponding side of the fixation (d, right).

Data Analysis

We examined whether the use of dimension-based retro-cues was influenced by perceptual interference by applying the accuracy (ACC), response time (RT), and *d*-prime scores [d' = Z (hit rate) - Z (false alarm rate)] as indices of the sensitivity to change detection; these were tested separately for color and orientation report trials using repeated measures ANOVA (analysis of variance) with cue type (neutral cue vs. valid cue) and interference condition (mask vs. no mask) as within-subject factors. A separate ANOVA was conducted for each dependent variable (ACC, RT, and *d*-prime). Significant interactions and main effects were decomposed using pairwise comparisons. Paired-samples *t*-tests and Bayes factor analysis were conducted to analyze differences in cue efficiency and to examine differences in cue efficiency between the interference conditions. A significance level of $p < 0.05$ was used for

all tests. The η_p^2 value was used as an estimator of the effect size for ANOVA, and Cohen's d was used as an estimator of the effect size. The *p*-values of follow-up comparisons were corrected using the false discovery rate (FDR) corrections.

Results

Accuracy

The results of the pilot experiment are shown in SFigure 2, which presents the accuracy rate for each condition for the color report trials (SFigure 2a) and the orientation report trials (SFigure 2b).

For the color report trials, the ANOVA indicated no significant interaction between the cue type and interference condition (F (1,23) = 1.451, p = 0.241, η_p^2 = 0.059) and no significant main effects of the interference condition (F (1,23) = 0.724, p = 0.404, η_p^2 = 0.031), but it indicated significant main effects of the cue type (F (1,23) = 10.979, p = 0.003, η_p^2 = 0.323). Follow-up paired-samples t-tests showed that the ACC was significantly higher under the valid cue condition than under the neutral cue condition in the trials with masks (t (23) = 2.729, p = 0.048, Cohen's d = 0.557, BF_{10} = 4.166). No significant difference was found between the mask and no-mask conditions for either the valid cue trials (t (23) = 0.277, p = 0.784, Cohen's d = 0.056, BF_{10} = 0.222) or the neutral cue trials (t (23) = 1.328, p = 0.263, Cohen's d = 0.271, BF_{10} = 0.468).

For the orientation report trials, the ANOVA indicated no significant interaction between the cue type and interference condition (F (1,23) = 0.102, p = 0.752, η_p^2 = 0.004) and no significant main effects of the interference condition (F (1,23) = 1.341, p = 0.259, η_p^2 = 0.055), whereas it indicated significant main effects of the cue type (F (1,23) = 8.464, p = 0.008, η_p^2 = 0.269). Follow-up paired-samples t-tests showed that the ACC score was significantly higher under the valid cue condition than under the neutral cue condition in trials without masks (t (23) = 2.950, p = 0.028, Cohen's d = 0.602, BF_{10} = 6.396), but this significant difference disappeared in the mask trials (t (23) = 1.304, p = 0.410, Cohen's d = 0.266, BF_{10} = 0.456). No significant difference was evident between the mask and no-mask conditions for either the valid cue trials (t (23) = 1.032, p = 0.417, Cohen's d = 0.211, BF_{10} = 0.346) or the neutral cue

trials (t (23) = 0.491, p = 0.628, Cohen's d = 0.100, BF_{10} = 0.240).

***d*-prime**

The average d-prime values for the color report trials and the orientation report trials are illustrated in SFigure 2c and SFigure 2d, respectively.

For the color report trials, the ANOVA indicated a similar result pattern as seen for the analysis of ACC, with no significant interaction between the cue type and interference condition (F (1,23) = 1.718, p = 0.203, η_p^2 = 0.069) and no significant main effects of the interference condition (F (1,23) = 0.767, p = 0.390, η_p^2 = 0.032), but significant main effects of the cue type (F (1,23) = 11.178, p = 0.003, η_p^2 = 0.327). Follow-up paired-samples t-tests showed that d' was significantly greater under the valid cue condition than under the neutral-cue condition in the mask trials (t (23) = 2.780, p = 0.044, Cohen's d = 0.568, BF_{10} = 4.598). No significant difference was detected between the mask and no-mask conditions for either the valid cue trials (t (23) = 0.253, p = 0.802, Cohen's d = 0.052, BF_{10} = 0.221) or the neutral cue trials (t (23) = 1.426, p = 0.223, Cohen's d = 0.291, BF_{10} = 0.524).

For the orientation report trials, the ANOVA indicated no significant interaction between the cue type and interference condition (F (1,23) = 0.060, p = 0.809, η_p^2 = 0.003) and no significant main effects of the interference condition (F (1,23) = 1.447, p = 0.241, η_p^2 = 0.059), but it revealed significant main effects of the cue type (F (1,23) = 7.061, p = 0.014, η_p^2 = 0.235). Follow-up paired-samples t-tests showed no significant difference between the d–prime values of each cue conditions in the no-mask trials (t (23) = 2.349, p = 0.112, Cohen's d = 0.480, BF_{10} = 2.082) or in the mask trials (t (23) = 1.422, p = 0.338, Cohen's d = 0.290, BF_{10} = 0.522). No significant difference was detected between the mask and no-mask conditions for either the valid cue trials (t (23) = 0.988, p = 0.444, Cohen's d = 0.202, BF_{10} = 0.333) or the neutral cue trials (t (23) = 0.596, p = 0.557, Cohen's d = 0.122, BF_{10} = 0.252).

Reaction time

The average reaction time for the color report trials and the orientation report trials are also illustrated in SFigure 2e and SFigure 2f, respectively.

For the color report trials, the ANOVA indicated no significant interaction between the cue type and interference condition (F (1,23) = 1.818, p = 0.191, η_p^2 = 0.073) and no

significant main effects of the interference condition (F (1,23) = 0.722, p = 0.404, η_p^2 = 0.030), but it indicated significant main effects of the cue type (F (1,23) = 29.250, p < 0.001, η_p^2 = 0.560). Follow-up paired-samples t-tests showed that the reaction time was significantly shorter under the valid cue condition than under the neutral cue condition for the mask condition (t (23) = 5.245, p < 0.001, Cohen's d = 1.071, BF_{10} = 929.681) and for the no-mask condition (t (23) = 4.068, p = 0.002, Cohen's d = 0.830, BF_{10} = 67.527). No significant difference was noted between the mask and no-mask conditions for either the valid cue trials (t (23) = 0.050, p = 0.960, Cohen's d = 0.010, BF_{10} = 0.215) or the neutral cue trials (t (23) = 1.295, p = 0.277, Cohen's d = 0.264, BF_{10} = 0.451).

For the orientation report trials, the ANOVA indicated no significant interaction between the cue type and interference condition (F (1,23) = 0.105, p = 0.749, η_p^2 = 0.005) and no significant main effects of the interference condition (F (1,23) = 1.205, p = 0.284, η_p^2 = 0.050), but it indicated significant main effects of the cue type (F (1,23) = 32.206, p < 0.001, η_p^2 = 0.583). Follow-up paired-samples t-tests showed that the reaction time was significantly shorter under the valid cue condition than under the neutral cue condition for the mask condition (t (23) = 3.777, p = 0.001, Cohen's d = 0.771, BF_{10} = 35.797) and for the no-mask condition (t (23) = 4.733, p < 0.001, Cohen's d = 0.966, BF_{10} = 295.989). No significant difference was detected between the mask and no-mask conditions for either the valid cue trials (t (23) = 0.658, p = 0.517, Cohen's d = 0.134, BF_{10} = 0.261) or the neutral cue trials (t (23) = 0.800, p = 0.576, Cohen's d = 0.163, BF_{10} = 0.287).

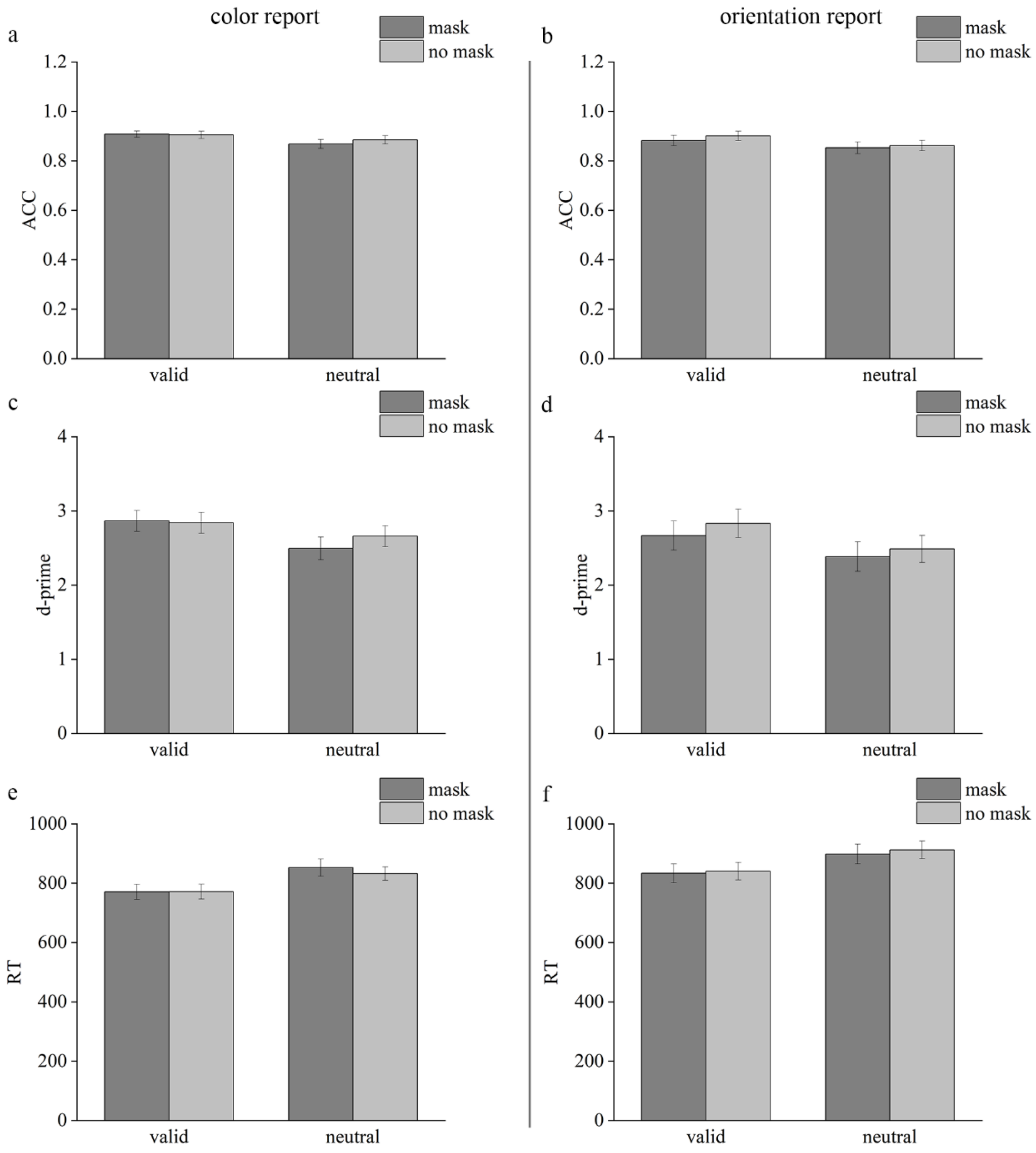

SFigure 2: ACC, *d*-prime, and RT results for the mask and no-mask conditions under each cue type condition in the color report and orientation report trials (n = 24). The dark gray bars represent means in the mask conditions. The light gray bars represent means in the no-mask conditions. Error bars represent within-subject SEMs.

Discussion

In this pilot study, we employed a change detection task and included a perceptual mask interference after retro-cues to disrupt the sustained attention to the cued dimension information. Our results, consistent with previous research (Hajonides et al., 2020; Heuer & Schubö, 2017; Niklaus et al., 2017; Park et al., 2017; Ye et al., 2016, 2021), showed a stable

dimension-based RCB across color and orientation report trials, reflected by higher ACC and *d*-prime values in the valid cue condition than in the neutral cue condition, suggesting that the participants did not neglect the retro-cues despite the low memory load. The RT was also shorter in the trials with valid cues than in the trials with neutral cues, indicating no accuracy-speed trade-off in the pilot experiment. Note that the RT results may have been influenced by participants' decision strategies, as we instructed them to respond as accurately as possible within a limited but adequate time.

Of particular interest, we did not observe any interaction effects between the cue type and interference condition, seemingly suggesting that prioritized dimension-based information does not demand sustained attention during the maintenance. However, we noticed a trend that the perceptual interference impacted the dimension-based RCB, as indicated by the ACC and *d*-prime values of orientation report trials, which showed the presence of the dimension-based RCB in the no-mask condition but its absence in the mask condition. In this pilot experiment, we selected stimuli from eight apparently different colors and orientations, resulting in the low precision of the change detection task. The change detection task indirectly tests the nature of VWM representations by requiring participants to compare stimuli and decide if they are identical or different. We cannot directly obtain the state of participants' representations, such as the concrete color or orientation in their VWM, from the ACC rate or other measures. Therefore, in our formal experiments in the main text, we used a recall task, which directly and more precisely tests the nature of VWM representations, to allow a better observation of the interference effects on the dimension-based RCB. Our results in the main text also show that recall tasks are more sensitive in detecting interference effects.

Another possible explanation for the absence of interaction effects in the pilot experiment is the potential invalidity or weakness of the masks, as the results showed no difference between the mask and no-mask conditions in both cue trials. To further explore this possibility, we conducted formal experiments using different types of interference or interruption.

The mixture model and swap model parameter results of Experiment 1

We fitted the data in Experiment 1 using the mixture model (Zhang & Luck, 2008) and the swap model (Bays et al., 2009) with the MemToolbox (Suchow et al., 2013). The mixture model assumes that participants' responses can be divided into two types of trials: those in which participants did not consolidate items into VWM, and instead guessed the information, conforming to a uniform random distribution, and those in which participants successfully consolidated the information into VWM, which contained a noisy representation of the target item, modeled by a von Mises distribution. The swap model assumes that the participants' behavior is a result of a mixture of three types of trials: in the first, the participants consolidated the items into VWM, containing a noisy representation of the target color or orientation, conforming to a von Mises distribution; in the second, the participants did not consolidate the items into VWM and instead guessed the reported color or orientation randomly, producing a uniform distribution; and in the third, the participants reported the non-target color or orientation during the response phase, resulting in a von Mises distribution of responses around the non-target. These models allowed us to estimate the guess rate, the precision of the memory representation (SD), and the non-target report rate.

Data Analysis

We explored the specific influenced component of the VWM performance when the use of dimension-based retro-cues was influenced by interfering information by applying the guess rate, SD, and non-target report rate as indices of the sensitivity to recall tasks. These dependent variables were tested separately for color and orientation report trials using repeated measures ANOVAs (analysis of variance) with cue type (neutral cue vs. valid cue) and interference condition (mask vs. no mask) as within-subject factors. A dependent ANOVA was conducted for each variable. Significant interactions and main effects were decomposed using pairwise comparisons. Paired-samples *t*-tests and Bayes factor analysis were conducted to

analyze differences in cue efficiency and to examine differences in cue efficiency between the mask and no-mask conditions. A significance level of $p < 0.05$ was used for all tests. The η_p^2 value was used as an estimator of the effect size for ANOVA, and the Cohen's d was used as an estimator of the effect size.

Results

Mixture model

Guess rate

The mixture model results of Experiment 1 are shown in SFigure 3, which presents the guess rate for each condition for the color report trials (SFigure 3a) and the orientation report trials (SFigure 3b).

For the color report trials, the ANOVA indicated no significant interaction between the cue type and interference condition (F (1,23) = 0.110, p = 0.744, η_p^2 = 0.005) and no significant main effects of the interference condition (F (1,23) = 0.018, p = 0.894, η_p^2 < 0.001), but it indicated significant main effects of the cue type (F (1,23) = 5.723, p = 0.025, η_p^2 = 0.199). Follow-up paired-samples t-tests showed that the guess rate was significantly lower under the valid cue condition than under the neutral cue condition in the mask trials (t (23) = 2.210, p = 0.037, Cohen’s d = 0.451, BF_{10} = 1.640). No significant difference was evident between the mask and no-mask conditions for either the valid cue trials (t (23) = 0.148, p = 0.884, Cohen’s d = 0.030, BF_{10} = 0.217) or the neutral cue trials (t (23) = 0.282, p = 0.780, Cohen’s d = 0.058, BF_{10} = 0.223).

For the orientation report trials, the ANOVA indicated no significant interaction between the cue type and interference condition (F (1,23) = 0.222, p = 0.642, η_p^2 = 0.010) and no significant main effects of the interference condition (F (1,23) = 2.693, p = 0.114, η_p^2 = 0.105) or the cue type (F (1,23) = 1.465, p = 0.238, η_p^2 = 0.060). Follow-up paired-samples t-tests showed no significant differences between the guess rate under the valid cue condition and the neutral cue condition in the mask trials (t (23) = 0.446, p = 0.660, Cohen’s d = 0.091, BF_{10} = 0.235) or in the no-mask trials (t (23) = 1.167, p = 0.255, Cohen’s d = 0.238, BF_{10} =

0.394). No significant difference was noted between the mask and no-mask conditions for either the valid cue trials (t (23) = 1.629, p = 0.117, Cohen's d = 0.332, BF_{10} = 0.679) or the neutral cue trials (t (23) = 0.742, p = 0.466, Cohen's d = 0.151, BF_{10} = 0.275).

SD

The average SD scores for the color report trials and the orientation report trials are illustrated in SFigure 3c and SFigure 3d, respectively.

For the color report trials, the ANOVA showed no significant interaction between the cue type and interference condition (F (1,23) = 0.040, p = 0.843, η_p^2 = 0.002) and no significant main effects of the interference condition (F (1,23) = 0.059, p = 0.810, η_p^2 = 0.003) or the cue type (F (1,23) = 0.227, p = 0.638, η_p^2 = 0.010). Follow-up paired-samples *t*-tests showed no significant differences between the SD under the valid cue condition and the neutral cue condition in the mask trials (t (23) = 0.371, p = 0.714, Cohen's d = 0.076, BF_{10} = 0.229) or in the no-mask trials (t (23) = 0.205, p = 0.839, Cohen's d = 0.042, BF_{10} = 0.219). No significant difference was apparent between the mask and no-mask conditions for either the valid cue trials (t (23) = 0.033, p = 0.974, Cohen's d = 0.007, BF_{10} = 0.215) or the neutral cue trials (t (23) = 0.298, p = 0.768, Cohen's d = 0.061, BF_{10} = 0.224).

For the orientation report trials, the ANOVA showed no significant interaction between the cue type and interference condition (F (1,23) = 0.863, p = 0.362, η_p^2 = 0.036) and no significant main effects of the interference condition (F (1,23) = 0.661, p = 0.425, η_p^2 = 0.028), but it revealed significant main effects of the cue type (F (1,23) = 5.534, p = 0.028, η_p^2 = 0.194). Follow-up paired-samples *t*-tests showed that the SD was significantly lower under the valid cue condition than under the neutral cue condition in the no-mask trials (t (23) = 2.885, p = 0.008, Cohen's d = 0.589, BF_{10} = 5.635), but this difference between cue types disappeared in the mask trials (t (23) = 0.856, p = 0.401, Cohen's d = 0.175, BF_{10} = 0.299). No significant difference was detected between the mask and no-mask conditions for either the valid cue trials (t (23) = 1.455, p = 0.159, Cohen's d = 0.297, BF_{10} = 0.543) or the neutral cue trials (t (23) = 0.084, p = 0.934, Cohen's d = 0.017, BF_{10} = 0.215).

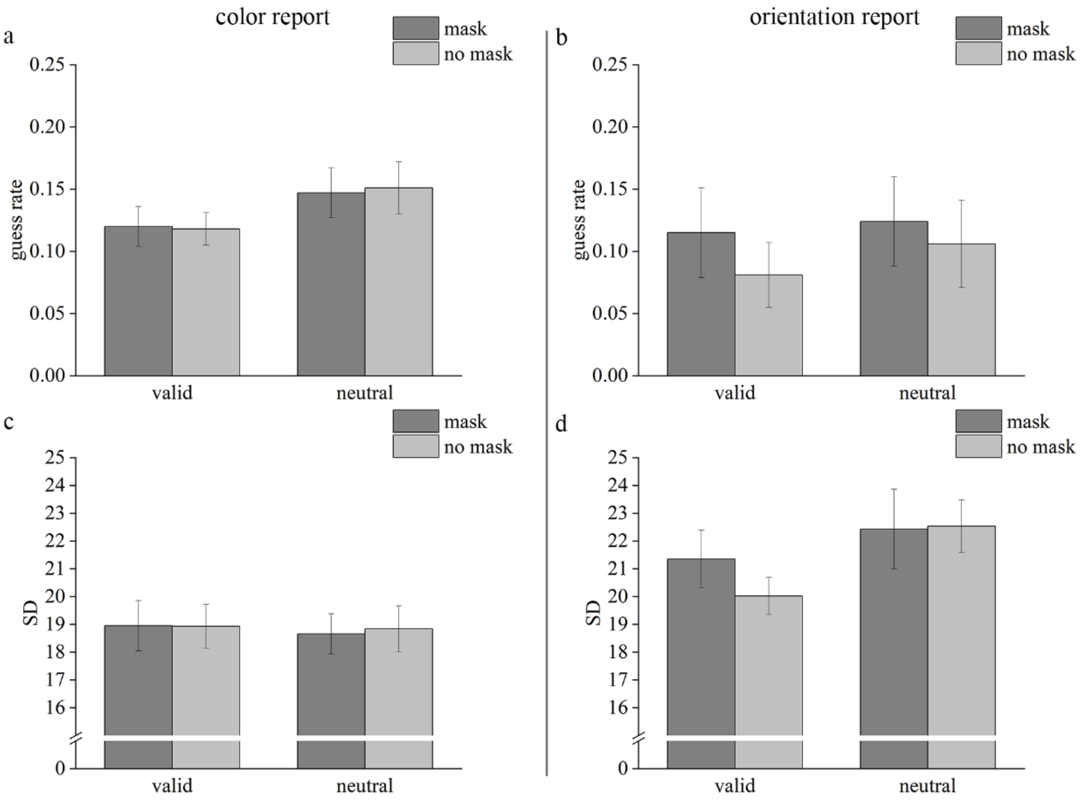

SFigure 3: The guess rate and SD results for the mask and no-mask conditions under each cue type condition in the color report and orientation report trials (n = 24) in Experiment 1. The dark gray bars represent means of the mask conditions. The light gray bars represent means of the no-mask conditions. Error bars represent within-subject SEMs.

Swap model

Guess rate

The swap model results of Experiment 1 are shown in SFigure 4, which presents the guess rate for each condition for the color report trials (SFigure 4a) and the orientation report trials (SFigure 4b).

For the color report trials, the ANOVA indicated a similar result pattern as seen for the analysis of guess rate of the mixture model, with no significant interaction between the cue type and interference condition (F (1,23) = 0.007, p = 0.932, η_p^2 < 0.001) and no significant main effects of the interference condition (F (1,23) = 0.032, p = 0.859, η_p^2 = 0.001) but significant main effects of the cue type (F (1,23) = 5.014, p = 0.035, η_p^2 = 0.179). Follow-up

paired-samples t-tests showed that the guess rate was significantly lower under the valid cue condition than under the neutral cue condition in the mask trials (t (23) = 2.222, p = 0.036, Cohen's d = 0.454, BF_{10} = 1.673). No significant difference was evident between the mask and no-mask conditions for either the valid cue trials (t (23) = 0.089, p = 0.929, Cohen's d = 0.018, BF_{10} = 0.215) or the neutral cue trials (t (23) = 0.168, p = 0.868, Cohen's d = 0.034, BF_{10} = 0.217).

For the orientation report trials, the ANOVA also indicated a similar result pattern to that observed for the analysis of guess rate of the mixture model, showing no significant interaction between the cue type and interference condition (F (1,23) = 0.533, p = 0.473, η_p^2 = 0.023) and no significant main effects of the interference condition (F (1,23) = 2.702, p = 0.114, η_p^2 = 0.105) or the cue type (F (1,23) = 0.314, p = 0.580, η_p^2 = 0.013). Follow-up paired-samples t-tests showed no significant differences between the guess rate under the valid cue condition and the neutral cue condition in the mask trials (t (23) = 0.681, p = 0.503, Cohen's d = 0.139, BF_{10} = 0.265) or in the no-mask trials (t (23) = 1.637, p = 0.115, Cohen's d = 0.334, BF_{10} = 0.687). No significant difference was detected between the mask and no-mask conditions for either the valid cue trials (t (23) = 1.951, p = 0.063, Cohen's d = 0.398, BF_{10} = 1.081) or the neutral cue trials (t (23) = 0.605, p = 0.551, Cohen's d = 0.123, BF_{10} = 0.253).

SD

The average SD scores for the color report trials and the orientation report trials are illustrated in SFigure 4c and SFigure 4d, respectively.

For the color report trials, the ANOVA indicated a similar result pattern as seen for the analysis of SD scores of mixture model, with no significant interaction between the cue type and interference condition (F (1,23) = 0.003, p = 0.958, η_p^2 < 0.001) and no significant main effects of the interference condition (F (1,23) = 0.035, p = 0.853, η_p^2 = 0.002) or the cue type (F (1,23) = 0.030, p = 0.864, η_p^2 = 0.001). Follow-up paired-samples t-tests showed no significant differences between the SD under the valid cue condition and the neutral cue condition in the mask trials (t (23) = 0.122, p = 0.904, Cohen's d = 0.025, BF_{10} = 0.216) or in the no-mask trials (t (23) = 0.099, p = 0.922, Cohen's d = 0.020, BF_{10} = 0.216). No significant

difference was detected between the mask and no-mask conditions for either the valid cue trials (t (23) = 0.155, p = 0.878, Cohen's d = 0.032, BF_{10} = 0.217) or the neutral cue trials (t (23) = 0.060, p = 0.953, Cohen's d = 0.012, BF_{10} = 0.215).

For the orientation report trials, the ANOVA also indicated a similar result pattern to that obtained for the analysis of SD scores of the mixture model, showing no significant interaction between the cue type and interference condition (F (1,23) = 0.622, p = 0.438, η_p^2 = 0.026) and no significant main effects of the interference condition (F (1,23) = 1.559, p = 0.224, η_p^2 = 0.063), but revealing significant main effects of the cue type (F (1,23) = 7.485, p = 0.012, η_p^2 = 0.246). Follow-up paired-samples t-tests showed that the SD was significantly lower under the valid cue condition than under the neutral cue condition in the no-mask trials (t (23) = 2.781, p = 0.011, Cohen's d = 0.568, BF_{10} = 4.605) but this difference between cue conditions disappeared in the mask trials (t (23) = 1.080, p = 0.292, Cohen's d = 0.220, BF_{10} = 0.361). No significant difference was evident between the mask and no-mask conditions for either the valid cue trials (t (23) = 1.910, p = 0.069, Cohen's d = 0.390, BF_{10} = 1.016) or the neutral cue trials (t (23) = 0.287, p = 0.777, Cohen's d = 0.059, BF_{10} = 0.223).

Non-target report rate

The average non-target report rates for the color report trials and the orientation report trials are also illustrated in SFigure 4e and SFigure 4f, respectively.

For the color report trials, the ANOVA indicated no significant interaction between the cue type and interference condition (F (1,23) = 0.285, p = 0.599, η_p^2 = 0.012) and no significant main effects of the interference condition (F (1,23) = 0.011, p = 0.919, η_p^2 < 0.001) or the cue type (F (1,23) = 0.314, p = 0.580, η_p^2 = 0.013). Follow-up paired-samples t-tests showed no significant differences between the non-target report rate under the valid cue condition and the neutral cue condition in the mask trials (t (23) = 0.064, p = 0.950, Cohen's d = 0.013, BF_{10} = 0.215) or in the no-mask trials (t (23) = 0.647, p = 0.524, Cohen's d = 0.132, BF_{10} = 0.260). No significant difference was observed between the mask and no-mask conditions for either the valid cue trials (t (23) = 0.576, p = 0.570, Cohen's d = 0.118, BF_{10} = 0.250) or the neutral cue trials (t (23) = 0.300, p = 0.767, Cohen's d = 0.061, BF_{10} = 0.224).

For the orientation report trials, the ANOVA indicated no significant interaction between

the cue type and interference condition (F (1,23) = 0.567, p = 0.459, η_p^2 = 0.024) and no significant main effects of the interference condition (F (1,23) = 0.230, p = 0.636, η_p^2 = 0.010) or the cue type (F (1,23) = 0.621, p = 0.439, η_p^2 = 0.026). Follow-up paired-samples t-tests showed no significant differences between the non-target report rate under the valid cue condition and the neutral cue condition in the mask trials (t (23) = 1.039, p = 0.310, Cohen's d = 0.212, BF_{10} = 0.348) or in the no-mask trials (t (23) = 1.637, p = 0.094, Cohen's d = 0.019, BF_{10} = 0.216). No significant difference was evident between the mask and no-mask conditions for either the valid cue trials (t (23) = 0.131, p = 0.897, Cohen's d = 0.027, BF_{10} = 0.216) or the neutral cue trials (t (23) = 1.244, p = 0.226, Cohen's d = 0.254, BF_{10} = 0.426).

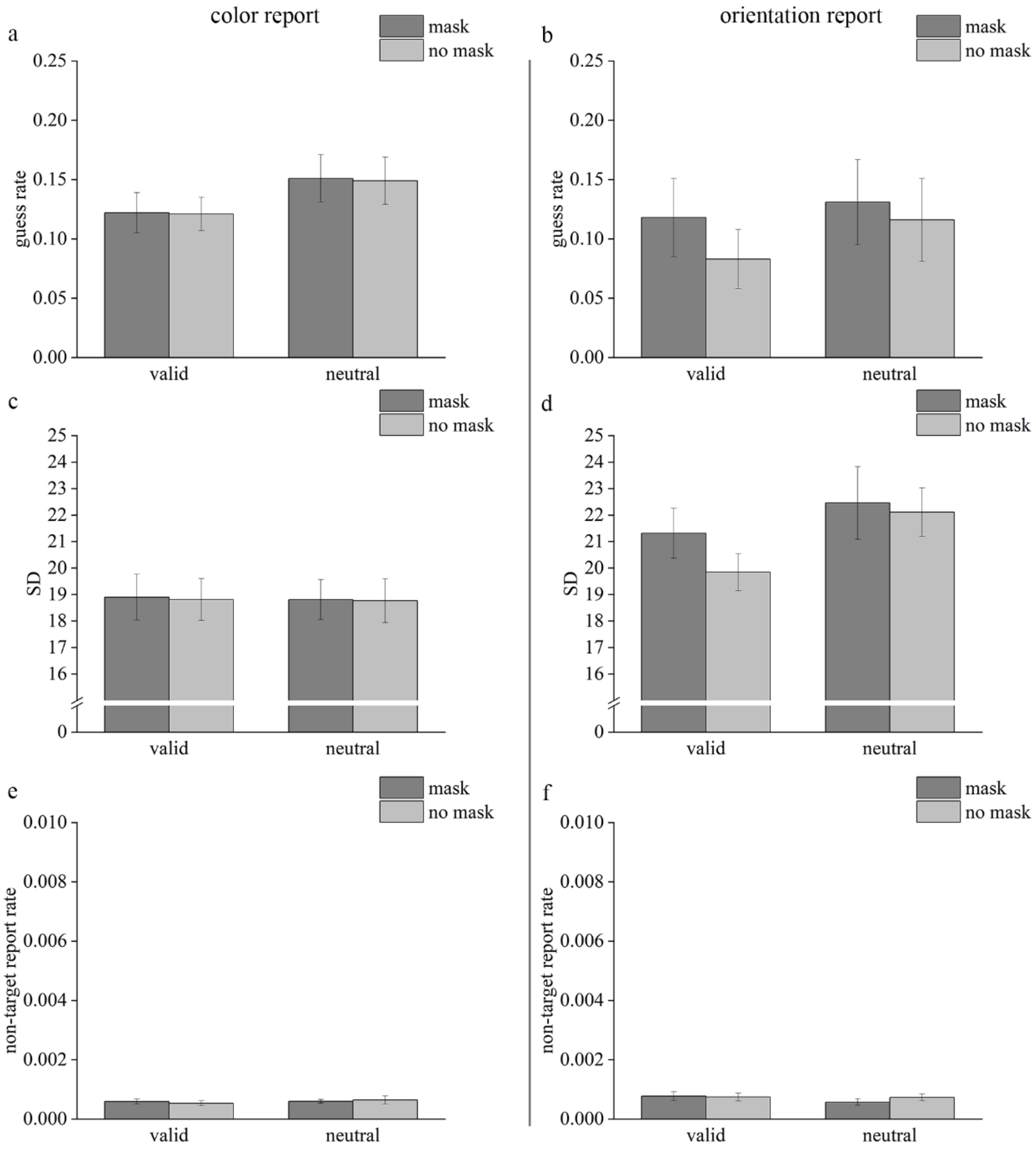

SFigure 4: The guess rate, SD, and non-target report rate results for the mask and no-mask conditions under each

cue type condition in the color report and orientation report trials (n = 24) in Experiment 1. The dark gray bars represent means of the mask conditions. The light gray bars represent means of the no-mask conditions. Error bars represent within-subject SEMs.

Discussion

Analyses of the results showed a consistent dimension-based RCB, with a lower guess rate for the color report trials and a higher SD for the orientation report trials in the valid retro-cue condition than in the neutral cue condition. These results aligned with previous findings by Ye et al. (2016) and demonstrated the differential impact of sustained attention on color and orientation VWM. Additionally, the low number of swap errors observed indicates that participants had a low probability of reporting non-target information.

Interestingly, we found a trend in which the masks had a stronger impact on the valid cue condition than the neutral cue condition. Our analyses of memory precision (SD) in the orientation report trials revealed that the dimension-based RCB was present only in the absence of masks and that it disappeared when sustained attention was disrupted by masks. This suggests that the interference during the post-cue maintenance phase impacted the interaction between sustained attention and VWM, leading to impaired improvement of dimension-based retro-cues. The lack of an interference effect in both the valid and neutral cue conditions weakened the impact of the interference—a finding that we further explored in Experiment 2 and Experiment 3.

We also observed a small dimension-based RCB for color VWM when sustained attention was disrupted by masks in Experiment 1, indicating that the sustained attention oriented toward target representations is not the only necessary component of the dimension-based RCB. However, in some situations, defocusing sustained attention may completely eliminate the dimension-based RCB. We further investigated the influence of different interference or interruption conditions on the dimension-based RCB by arranging a different cue-and-interference/interruption SOA in Experiments 2 & 4 and using an odd-even task as interruption in Experiments 3 & 4. In addition, the reduced number of trials in each condition in Experiment 2 (64 trials for each condition) and Experiments 3 & 4 (32 or 64 trials for each

condition) meant that the number of trials was insufficient to fit the mixture and swap models to the data.

Results of the circular standard deviation of error in four experiments

We subtracted the value of the tested dimension from the report value that participants chose and calculated the circular standard deviation of these errors (SD_{error}) for each participant and each experimental condition in the four experiments. We investigated the influence of the interference or interruption on the use of dimension-based retro-cues by conducting repeated measures ANOVA for the SD_{error} with different cue types (neutral cue vs. valid cue) and interference/interruption conditions (mask vs. no mask; or dual task vs. single task) as within-subject factors. Two-tailed *t*-tests were conducted for follow-up comparisons between different conditions. A significance level of $p < 0.05$ was used for all tests. The η_p^2 value was used as an estimator of the effect size for ANOVA. The Cohen's d was used as an estimator of the effect size. A Bayes factor analysis was also used to report the *t*-test results.

The analyses of SD_{error} showed similar result patterns to those of the offsets across the four experiments. Therefore, we transferred the SD_{error} results to the Supplementary Materials to simplify the formal manuscript.

Results

Experiment 1

SFigure 5 shows the SD_{error} results of Experiment 1 for each condition for the color report trials (SFigure 5a) and the orientation report trials (SFigure 5b).

For the color report trials, the ANOVA indicated no significant interaction between the cue type and interference condition (F (1,23) = 0.007, p = 0.935, $\eta_p^2 < 0.001$) and no significant main effects of the interference condition (F (1,23) = 0.773, p = 0.388, $\eta_p^2 = 0.033$)

or of the cue type (F (1,23) = 3.626, p = 0.069, η_p^2 = 0.136). Follow-up paired-samples t-tests showed no significant difference between the cue type conditions for either the mask trials (t (23) = 1.964, p = 0.062, Cohen's d = 0.401, BF_{10} = 1.103) or the no-mask trials (t (23) = 1.307, p = 0.204, Cohen's d = 0.267, BF_{10} = 0.457). No significant difference was revealed between the mask and no-mask conditions for either the valid cue trials (t (23) = 0.635, p = 0.531, Cohen's d = 0.130, BF_{10} = 0.258) or the neutral cue trials (t (23) = 0.542, p = 0.593, Cohen's d = 0.111, BF_{10} = 0.245).

For the orientation report trials, the ANOVA indicated significant interaction between the cue type and interference condition (F (1,23) = 6.552, p = 0.018, η_p^2 = 0.222) and significant main effects of the interference condition (F (1,23) = 16.535, p < 0.001, η_p^2 = 0.418) and of the cue type (F (1,23) = 15.326, p < 0.001, η_p^2 = 0.400). Follow-up paired-samples t-tests showed that the SD_{error} score was significantly smaller under the valid cue condition than under the neutral cue condition in the mask trials (t (23) = 2.428, p = 0.023, Cohen's d = 0.496, BF_{10} = 2.392) and in the no-mask trials (t (23) = 4.694, p < 0.001, Cohen's d = 0.958, BF_{10} = 271.208). The SD_{error} was significantly larger in the mask condition than in the no-mask condition for the valid cue trials (t (23) = 5.656, p < 0.001, Cohen's d = 1.154, BF_{10} = 2316.297), but this difference between interference conditions disappeared in the neutral cue trials (t (23) = 1.604, p = 0.122, Cohen's d = 0.327, BF_{10} = 0.657).

Experiment 2

SFigure 5 shows the SD_{error} results for Experiment 2 for each condition for the color report trials (SFigure 5c) and the orientation report trials (SFigure 5d).

For the color report trials, the ANOVA indicated a significant interaction between the cue type and interference condition (F (1,22) = 7.941, p = 0.010, η_p^2 = 0.265) and significant main effects of the interference condition (F (1,22) = 8.568, p = 0.008, η_p^2 = 0.280) and of the cue type (F (1,22) = 16.283, p < 0.001, η_p^2 = 0.425). Follow-up paired-samples t-tests showed that the performance was significantly better in the valid cue condition than in the neutral cue condition for the no-mask trials (t (22) = 4.878, p < 0.001, Cohen's d = 1.017, BF_{10} = 371.070), but this improvement disappeared in the mask trials (t (22) = 1.401, p = 0.175,

Cohen's d = 0.292, BF_{10} = 0.517). The SD_{error} was significantly larger in the mask condition than in the no-mask condition for the valid cue trials (t (22) = 3.830, p < 0.001, Cohen's d = 0.799, BF_{10} = 38.411), but no difference existed between interference conditions for the neutral cue trials (t (22) = 0.097, p = 0.924, Cohen's d = 0.020, BF_{10} = 0.220).

For the orientation report trials, the ANOVA indicated no significant interaction between the cue type and interference condition (F (1,22) = 2.716, p = 0.114, η_p^2 = 0.110), but significant main effects of the interference condition (F (1,22) = 5.839, p = 0.024, η_p^2 = 0.210) and of the cue type (F (1,22) = 4.643, p = 0.042, η_p^2 = 0.174). Follow-up paired-samples *t*-tests showed that the performance was significantly better in the valid cue condition than in the neutral cue conditions for the no-mask trials (t (22) = 2.506, p = 0.020, Cohen's d = 0.522, BF_{10} = 2.748), but this improvement disappeared in the mask trials (t (22) = 0.180, p = 0.859, Cohen's d = 0.038, BF_{10} = 0.222). The SD_{error} was significantly larger in the mask condition than in the no-mask condition for the valid cue trials (t (22) = 2.839, p = 0.010, Cohen's d = 0.592, BF_{10} = 5.104), but no difference existed between interference conditions for the neutral cue trials (t (22) = 0.494, p = 0.626, Cohen's d = 0.103, BF_{10} = 0.244).

Experiment 3

SFigure 5 shows the SD_{error} results for Experiment 3 for each condition for the color report trials (SFigure 5e) and the orientation report trials (SFigure 5f).

For the color report trials, the ANOVA indicated a significant interaction between the cue type and interruption condition (F (1,22) = 8.230, p = 0.009, η_p^2 = 0.272) and significant main effects of the interruption condition (F (1,22) = 16.886, p < 0.001, η_p^2 = 0.434) and of the cue type (F (1,22) = 23.195, p < 0.001, η_p^2 = 0.513). Follow-up paired-samples *t*-tests showed that the performance was significantly better in valid cue condition than in neutral cue conditions for the single task trials (t (22) = 4.955, p < 0.001, Cohen's d = 1.033, BF_{10} = 439.091), but this improvement disappeared in the dual task trials (t (22) = 1.948, p = 0.064, Cohen's d = 0.406, BF_{10} = 1.085). The SD_{error} was significantly larger in the dual task condition than in the single task condition for the valid cue trials (t (22) = 5.853, p < 0.001, Cohen's d = 1.220, BF_{10} = 3065.329), but no difference existed between the interruption conditions for the

neutral cue trials (t (22) = 0.423, p = 0.676, Cohen's d = 0.088, BF_{10} = 0.237).

For the orientation report trials, the ANOVA indicated no significant interaction between the cue type and interruption condition (F (1,22) = 2.043, p = 0.167, η_p^2 = 0.085), but significant main effects of the interruption condition (F (1,22) = 10.104, p = 0.004, η_p^2 = 0.315) and of the cue type (F (1,22) = 18.420, p < 0.001, η_p^2 = 0.456). Follow-up paired-samples t-tests showed that the performance was significantly better in the valid cue condition than in the neutral cue conditions for both the single task trials (t (22) = 3.532, p = 0.002, Cohen's d = 0.736, BF_{10} = 20.488) and the dual task trials (t (22) = 3.733, p = 0.001, Cohen's d = 0.778, BF_{10} = 31.226). The SD_{error} was significantly larger in the dual task condition than in the single task condition for the valid cue trials (t (22) = 3.196, p = 0.004, Cohen's d = 0.666, BF_{10} = 10.285), but no difference existed between interruption conditions for the neutral cue trials (t (22) = 1.459, p = 0.159, Cohen's d = 0.304, BF_{10} = 0.554).

Experiment 4

SFigure 5 shows the SD_{error} results for Experiment 4 for each condition for the color report trials (SFigure 5g) and the orientation report trials (SFigure 5h).

For the color report trials, the ANOVA indicated a significant interaction between the cue type and interruption condition (F (1,23) = 7.358, p = 0.012, η_p^2 = 0.242) and significant main effects of the interruption condition (F (1,23) = 22.394, p < 0.001, η_p^2 = 0.493) and of the cue type (F (1,23) = 12.302, p = 0.002, η_p^2 = 0.348). Follow-up paired-samples t-tests showed that the performance was significantly better in valid cue condition than in neutral cue conditions for the single task trials (t (23) = 3.554, p = 0.002, Cohen's d = 0.725, BF_{10} = 22.164), but this improvement disappeared in the dual task trials (t (23) = 1.473, p = 0.154, Cohen's d = 0.301, BF_{10} = 0.555). The SD_{error} was significantly larger in the dual task condition than in the single task condition for the valid cue trials (t (23) = 4.591, p < 0.001, Cohen's d = 0.937, BF_{10} = 215.630), but no difference existed between the interruption conditions for the neutral cue trials (t (23) = 1.464, p = 0.157, Cohen's d = 0.299, BF_{10} = 0.550).

For the orientation report trials, the ANOVA indicated a significant interaction between the cue type and interruption condition (F (1,23) = 5.591, p = 0.027, η_p^2 = 0.196) and

significant main effects of both the interruption condition (F (1,23) = 17.093, p < 0.001, η_p^2 = 0.426) and the cue type (F (1,23) = 22.112, p < 0.001, η_p^2 = 0.490). Follow-up paired-samples t-tests showed a comparable tendency of results for orientation to for color report trials, revealing that the performance was significantly better in valid cue condition than in neutral cue conditions for the single task trials (t (23) = 4.242, p < 0.001, Cohen's d = 0.866, BF_{10} = 99.183), but this improvement disappeared in the dual task trials (t (23) = 1.566, p = 0.131, Cohen's d = 0.320, BF_{10} = 0.625). The SD_{error} was significantly larger in the dual task condition than in the single task condition for the valid cue trials (t (23) = 6.066, p < 0.001, Cohen's d = 1.238, BF_{10} = 5720.861), but no difference existed between interruption conditions for the neutral cue trials (t (23) = 0.927, p = 0.363, Cohen's d = 0.189, BF_{10} = 0.316).

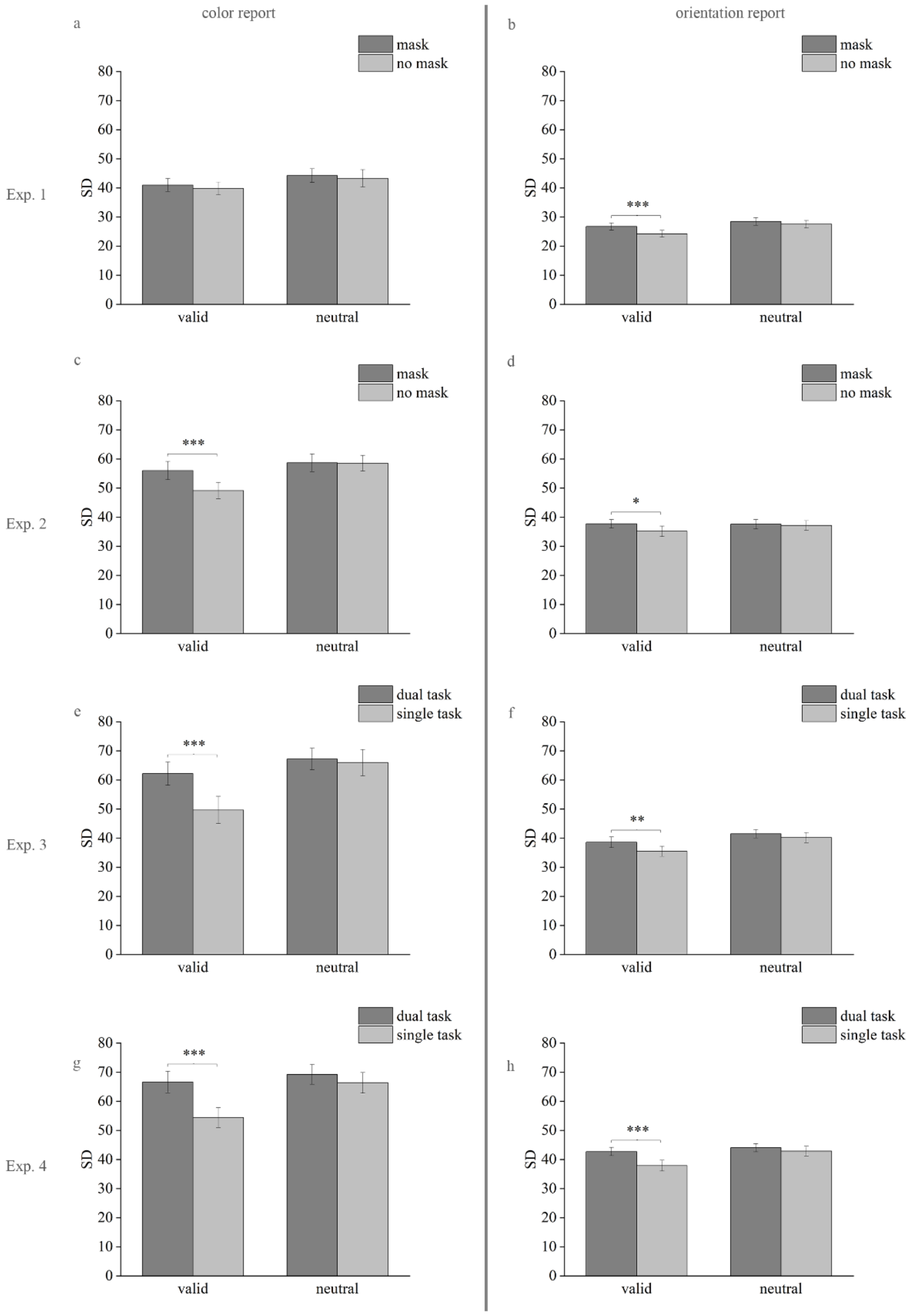

SFigure 5: The SD_{error} results for each interference/interruption condition with each cue type in the color report and orientation report trials in the four experiments. The dark gray bars represent means of the mask or the dual task condition. The light gray bars represent means of the no-mask or the single task condition. Error bars represent within-subject SEMs.

Discussion

In our study, we conducted four experiments to examine the impact of interference or interruption which was inserted into different stage of the prioritization of cued representation on the dimension-based RCB in VWM. We employed masks or cognitive tasks to suspend the sustained attention to the retro-cued dimension in each experiment. Based on the SD_{error} results, we found that interference or interruption impaired the memory performance of the valid-cued dimension, but they did not affect the VWM of neutral-cued trials, except for the color report trials in Experiment 1. These results highlight the greater susceptibility of the retro-cued dimension in VWM to interference or interruption compared to the dimension with normal attention allocation. Furthermore, we observed that the dimension-based RCB was eliminated by a mask or a secondary task in Experiments 2, 3 (for the color report trials) and 4, indicating the requirement of sustained attention to retro-cued dimension in VWM, across two stages of prioritization process, for the dimension-based RCB.

Throughout all four experiments, we observed a consistent pattern of smaller SD_{error} values for valid cue trials than for neutral cue trials in the absence of interference, except for the color report trials in Experiment 1. This pattern is consistent with previous research on the dimension-based RCB (Hajonides et al., 2020; Heuer & Schubö, 2017; Niklaus et al., 2017; Park et al., 2017; Ye et al., 2016, 2021), and suggests that retro-cues effectively enhance VWM performance. Note that the addition of masks or secondary tasks in the valid cue conditions across the four experiments impaired VWM performance, indicating that participants did not ignore the interference or interruption. In Experiments 3 and 4, we confirmed that participants' attention was drawn to the interruption by requiring them to maintain visual fixation on it and excluding trials with errors in the odd-even task from subsequent analyses.

Despite the transfer of attention from the cued dimension in VWM during the maintenance phase, the presence of the dimension-based RCB for the orientation report trials in Experiments 1 and 3 suggests that sustained attention on the target dimension is not the sole component necessary for the dimension-based RCB. Conversely, the absence of the dimension-based RCB in some conditions indicates that disruption of sustained attention does

impair the dimension-based RCB, which is regulated by a temporal factor. Specifically, when the cue-and-interference/interruption SOA was short (e.g., 400 ms in Experiment 2 and 550 ms in Experiment 4), attentional deployment directed by the retro-cue was not completed, and attention was partially drawn to the interference or interruption, leading to faults in the prioritization of the cued dimension information.

The distribution of reported colors as a function of the actual colors

In our study, we utilized a color wheel composed of colors that linearly changed in saturation (RGB) at each step, following the methodology in Ye et al. (2016). The RGB value was assigned as follows:

[255, 75 + 3 × n, 75] for < = 0 n < = 59

[255 − 3 × (n − 60), 255, 75] for 60 < = n < = 119

[75, 255, 75 + 3 × (n − 120)] for 120 < = n < = 179

[75, 255 – 3 × (n − 180), 255] for 180 < = n < = 239

[75 + 3 × (n − 240), 75, 255] for 240 < = n < = 299

[255, 75, 255 – 3 × (n − 300)] for 300 < = n < = 359

This experimental setup may elicit variations in the salience of the color stimuli and potentially impact the participants' color selection decisions. It is possible that the participants categorized the continuous color values into prototypes (e.g., red, yellow, blue, green, purple). To evaluate this possibility, we pooled the data from each experiment rather than for each participant, as color categories are highly consistent across individuals from a restricted age range and cultural group. We then plotted the distribution of the reported colors as a function of the actual colors for this pool of participants (see SFigure 6a for Experiment 1, SFigure 6b for Experiment 2, SFigure 6c for Experiment 3 and SFigure 6d for Experiment 4). If the participants represented the actual color (plus noise), then function should yield a straight line. Alternatively, if the participants represented a given sample color as the nearest color category value, the function should have a staircase appearance, in which variations in the actual color

within a given range lead to no change in the reported color, with a sudden change in reported color when the actual color crosses the category boundary. Our results, presented in SFigure 6, clearly follow a straight line with no sign of staircase-like horizontal bands, indicating that our participants remembered the actual color rather than the nearest color prototype.

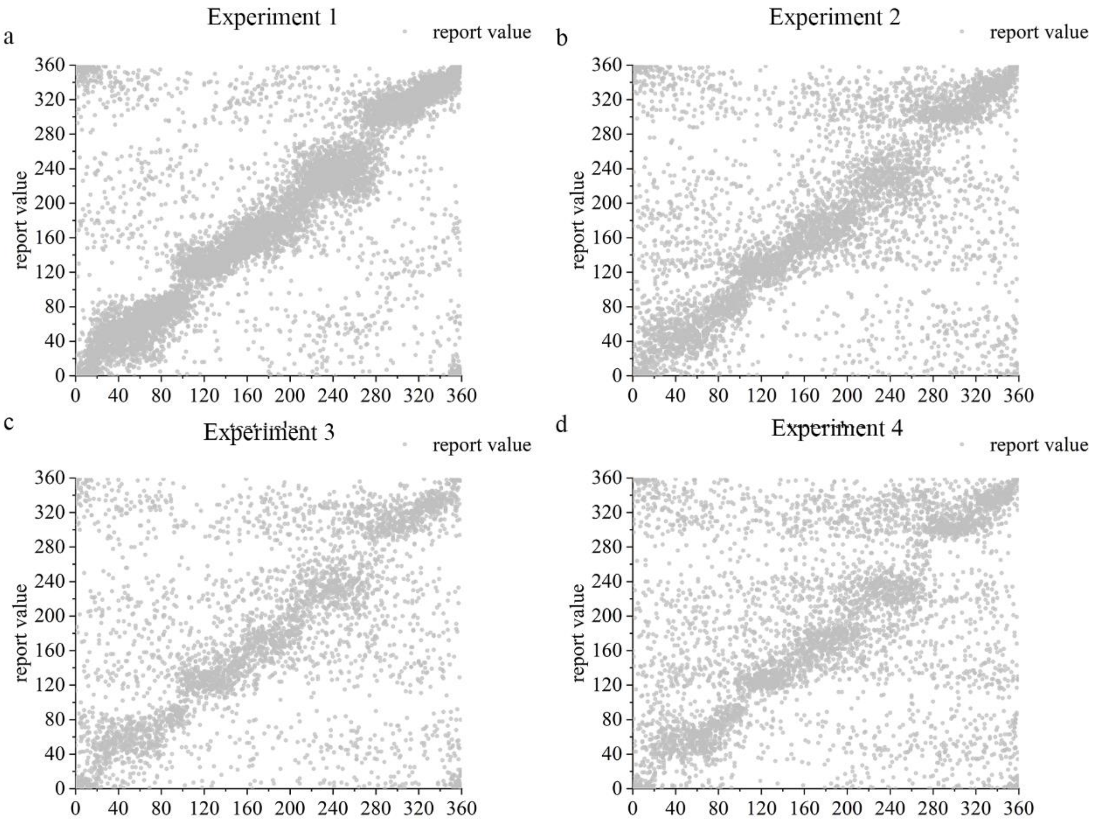

SFigure 6: Distribution of reporting a given color value as a function of the actual color value for (a) Experiment 1, (b) Experiment 2, (c) Experiment 3 and (d) Experiment 4, aggregated across at least 129 trials for each participant in each experiment.

We observed that participants exhibited a lower likelihood of selecting colors located near two specific values on the color wheel (i.e., green with RGB values of 75, 255, 75 and purple with RGB values of 195, 75, 255). This finding may suggest that certain colors with low luminance are difficult to remember than others. Alternatively, the location of these colors may be awkward to select with a mouse, leading to participants avoiding these values in their color selections.

It should be noted that this influence of the color wheel setting constitutes a systematic error in our study. However, we believe that this error is unlikely to have a significant impact

on the within-subject comparison conducted in our study. Furthermore, no similar systematic errors were evident in the orientation report trials, indicating that this additional analysis should not impact the overall conclusions drawn in the main text.

Reference

Bays, P. M., Catalao, R. F. G., & Husain, M. (2009). The precision of visual working memory is set by allocation of a shared resource. *Journal of Vision*, *9*(10), 7–7. https://doi.org/10.1167/9.10.7

Faul, F., Erdfelder, E., Lang, A.-G., & Buchner, A. (2007). G*Power 3: A flexible statistical power analysis program for the social, behavioral, and biomedical sciences. *Behavior Research Methods*, *39*(2), 175–191. https://doi.org/10.3758/BF03193146

Hajonides, J. E., van Ede, F., Stokes, M. G., & Nobre, A. C. (2020). Comparing the prioritization of items and feature-dimensions in visual working memory. *Journal of Vision*, *20*(8), 25. https://doi.org/10.1167/jov.20.8.25

Heuer, A., & Schubö, A. (2017). Selective weighting of action-related feature dimensions in visual working memory. *Psychonomic Bulletin & Review*, *24*(4), 1129–1134. https://doi.org/10.3758/s13423-016-1209-0

Hollingworth, A., & Maxcey-Richard, A. M. (2013). Selective maintenance in visual working memory does not require sustained visual attention. *Journal of Experimental Psychology: Human Perception and Performance*, *39*(4), 1047–1058. https://doi.org/10.1037/a0030238

Janczyk, M., & Berryhill, M. E. (2014). Orienting attention in visual working memory requires central capacity: Decreased retro-cue effects under dual-task conditions. *Attention, Perception, & Psychophysics*, *76*(3), 715–724. https://doi.org/10.3758/s13414-013-0615-x

Makovsik, T., & Jiang, Y. V. (2007). Distributing versus focusing attention in visual short-term memory. *Psychonomic Bulletin & Review*, *14*(6), 1072–1078. https://doi.org/10.3758/bf03193093

Niklaus, M., Nobre, A. C., & van Ede, F. (2017). Feature-based attentional weighting and spreading in visual working memory. *Scientific Reports*, *7*(1), 42384. https://doi.org/10.1038/srep42384

Park, Y. E., Sy, J. L., Hong, S. W., & Tong, F. (2017). Reprioritization of Features of Multidimensional Objects Stored in Visual Working Memory. *Psychological Science*, *28*(12), 1773–1785. https://doi.org/10.1177/0956797617719949

Rerko, L., Souza, A. S., & Oberauer, K. (2014). Retro-cue benefits in working memory without sustained focal attention. *Memory & Cognition*, *42*(5), 712–728. https://doi.org/10.3758/s13421-013-0392-8

Suchow, J. W., Brady, T. F., Fougnie, D., & Alvarez, G. A. (2013). Modeling visual working memory with the MemToolbox. *Journal of Vision*, *13*(10), 9. https://doi.org/10.1167/13.10.9

van Moorselaar, D., Gunseli, E., Theeuwes, J., & N. L. Olivers, C. (2015). The time course of protecting a visual memory representation from perceptual interference. *Frontiers in Human Neuroscience*, *8*. https://doi.org/10.3389/fnhum.2014.01053

Ye, C., Hu, Z., Ristaniemi, T., Gendron, M., & Liu, Q. (2016). Retro-dimension-cue benefit in visual working memory. *Scientific Reports*, *6*(1), 35573. https://doi.org/10.1038/srep35573

Ye, C., Xu, Q., Liu, X., Astikainen, P., Zhu, Y., Hu, Z., & Liu, Q. (2021). Individual differences in working memory capacity are unrelated to the magnitudes of retrocue benefits. *Scientific Reports*, *11*(1), 7258. https://doi.org/10.1038/s41598-021-86515-5

Zhang, W., & Luck, S. J. (2008). Discrete fixed-resolution representations in visual working

memory. *Nature*, *453*(7192), 233–235. https://doi.org/10.1038/nature06860
